# Interventions to improve oral vaccine performance: a systematic review and meta-analysis

**DOI:** 10.1016/S1473-3099(18)30602-9

**Published:** 2019-02

**Authors:** James A Church, Edward P Parker, Beth D Kirkpatrick, Nicholas C Grassly, Andrew J Prendergast

**Affiliations:** a Centre for Genomics & Child Health, Blizard Institute, Queen Mary University of London, London, UK; b Zvitambo Institute for Maternal and Child Health Research, Harare, Zimbabwe; c Department of Infectious Disease Epidemiology, St Mary's Campus, Imperial College London, London, UK; d Department of Microbiology and Molecular Genetics, Vaccine Testing Center, University of Vermont College of Medicine, Burlington, VT, USA

## Abstract

**Background:**

Oral vaccines underperform in low-income and middle-income countries compared with in high-income countries. Whether interventions can improve oral vaccine performance is uncertain.

**Methods:**

We did a systematic review and meta-analysis of interventions designed to increase oral vaccine efficacy or immunogenicity. We searched Ovid-MEDLINE and Embase for trials published until Oct 23, 2017. Inclusion criteria for meta-analysis were two or more studies per intervention category and available seroconversion data. We did random-effects meta-analyses to produce summary relative risk (RR) estimates. This study is registered with PROSPERO (CRD42017060608).

**Findings:**

Of 2843 studies identified, 87 were eligible for qualitative synthesis and 66 for meta-analysis. 22 different interventions were assessed for oral poliovirus vaccine (OPV), oral rotavirus vaccine (RVV), oral cholera vaccine (OCV), and oral typhoid vaccines. There was generally high heterogeneity. Seroconversion to RVV was significantly increased by delaying the first RVV dose by 4 weeks (RR 1·37, 95% CI 1·16–1·62) and OPV seroconversion was increased with monovalent or bivalent OPV compared with trivalent OPV (RR 1·51, 95% CI 1·20–1·91). There was some evidence that separating RVV and OPV increased RVV seroconversion (RR 1·21, 95% CI 1·00–1·47) and that higher vaccine inoculum improved OCV seroconversion (RR 1·12, 95% CI 1·00–1·26). There was no evidence of effect for anthelmintics, antibiotics, probiotics, zinc, vitamin A, withholding breastfeeding, extra doses, or vaccine buffering.

**Interpretation:**

Most strategies did not improve oral vaccine performance. Delaying RVV and reducing OPV valence should be considered within immunisation programmes to reduce global enteric disease. New strategies to address the gap in oral vaccine efficacy are urgently required.

**Funding:**

Wellcome Trust, Bill & Melinda Gates Foundation, UK Medical Research Council, and WHO Polio Research Committee.

## Introduction

Despite global declines in enteric disease, approximately 650 000 children die from diarrhoea annually,^1^ with rotavirus causing a third of deaths.^2^ A major obstacle to sustained progress is the reduced efficacy of oral vaccines in low-income and middle-income countries (LMICs).^3^ This phenomenon was first observed for oral poliovirus vaccine (OPV) in the 1950s and has since been described for several oral vaccines in many countries. For example, rotavirus vaccine (RVV) efficacy against severe rotavirus gastroenteritis is only 39% in sub-Saharan Africa^4^ and 48% in south Asia,^5^ compared with 85–98% in Europe and the USA.^6^, ^7^ The reasons for oral vaccine underperformance are unclear.^8^ Potential explanations include concurrent enteric infections,^9^, ^10^ microbiota composition,^11^ environmental enteric dysfunction,^12^ interference from maternal antibodies,^13^, ^14^ histoblood group antigens,^15^ and micronutrient deficiencies.^16^, ^17^ The relative contributions of these factors can vary depending on oral vaccine target, recipient age, and setting.

Multiple studies have assessed interventions to improve oral vaccine performance, broadly categorised as adjunctive interventions given before or with vaccination (such as micronutrient supplements or antimicrobials) or adjustments to the vaccine formulation and delivery schedule (such as increased vaccine inoculum or altered timing). Reviews have assessed specific interventions^18^ or individual vaccines,^19^, ^20^ but none have assessed the full range of approaches used across all oral vaccines. We therefore did a systematic review and meta-analysis of interventions to increase oral vaccine efficacy or immunogenicity in LMICs.

## Methods

### Search strategy and selection criteria

We followed PRISMA guidelines throughout our review. We searched Ovid-MEDLINE and Embase for English language articles published any time up to Oct 23, 2017, describing studies assessing interventions to improve oral vaccine performance (appendix p 2). We examined the reference lists of articles to identify additional studies, searched the grey literature, and contacted experts for unpublished data.

We screened full-text papers for inclusion in the qualitative synthesis. We included randomised trials, cluster-randomised trials, non-randomised trials, and meta-analyses assessing one or more interventions. We did not include case-control studies, controlled before–after studies, or observational data from cross-sectional studies and case series. Studies were excluded if they included fewer than ten participants, did not include a control group, or did not measure vaccine efficacy or immunogenicity. We excluded strategies bypassing the oral route (eg, use of inactivated poliovirus vaccine), and pre-licensure dose-finding trials. We did not exclude studies on the basis of participant age or setting, because studies from high-income countries or in older age groups might provide insights into oral vaccine failure among infants in LMICs. For meta-analysis, inclusion criteria required two or more studies per intervention category and available seroconversion data. The protocol is available at www.crd.york.ac.uk/PROSPERO, CRD42017060608.

**Research in context**

**Evidence before this study**

Oral vaccines consistently underperform when given to children in low-income and middle-income countries (LMICs) but the underlying causes and potential intervention approaches are unclear. We searched Ovid-MEDLINE and Embase for systematic reviews and meta-analyses of interventions to improve oral vaccine performance published up to May 31, 2018, using the same search strategy detailed in the appendix (p 5). We identified three systematic reviews, one including a meta-analysis. The meta-analysis, published in 1998, found that regimens containing a single dose of typhoid vaccine were less effective than were regimens with two or more doses. Of the remaining two systematic reviews, both published in 2017, one examined differences in rotavirus vaccine scheduling across eight trials, concluding that seroconversion was lower among children given the vaccine earlier in infancy (age 6 and 10 weeks) versus later in infancy (age 10 and 14 weeks). The other, restricted to probiotics, found a beneficial effect of probiotics on vaccine responses (parenteral and oral vaccines) in half of the studies. However, there have been no systematic reviews assessing all intervention strategies (both adjunctive and vaccine design or delivery adjustments) across oral poliovirus, rotavirus, cholera, and typhoid vaccines. We assessed the full range of intervention approaches and oral vaccines investigated to date, to identify whether any strategies could be adopted by immunisation programmes, and to identify research gaps to inform future trials.

**Added value of this study**

This is the first systematic review and meta-analysis of approaches to improve oral vaccine performance among children. We assessed the evidence for 22 interventions targeting four oral vaccines. Overall, we found few interventions had a substantial benefit on the basis of the available evidence, highlighting the challenge in overcoming oral vaccine underperformance. However, we found that delaying the first dose of rotavirus vaccine and reducing oral poliovirus vaccine valence can improve oral vaccine immunogenicity.

**Implications of all the available evidence**

Existing oral vaccines and their schedules are poorly effective among children in LMICs. Most adjunctive interventions to date have not improved oral vaccine performance. Untested interventions such as water, sanitation and hygiene, the effect of booster doses given later in infancy, and increasing vaccine inoculum for rotavirus vaccine warrant further study. Cost-benefit and modelling analyses that consider the full effect of delaying the first dose of rotavirus vaccine should be undertaken. However, the global research community should also strongly consider new and innovative ways to address this efficacy gap, including a decreased reliance on oral vaccines, to reduce the global burden of enteric disease.

### Outcome definitions

The prespecified primary outcome was oral vaccine performance, defined as either vaccine efficacy or immunogenicity, depending on study design. Vaccine efficacy was defined as percentage disease reduction in the vaccinated group compared with the unvaccinated group. Our prespecified preferred measure of vaccine immunogenicity was the proportion of children with seroconversion, as defined by each study, after the last scheduled vaccine dose. Alternative measures included geometric mean titres and fold-rise if seroconversion was not reported (appendix p 8). Studies without seroconversion data were included in the qualitative synthesis but were not eligible for meta-analysis. Timing of vaccine immunogenicity measurement was not considered in the eligibility criteria. The chosen correlates of protection were serum neutralising antibodies for OPV, serum IgA for RVV, and vibriocidal antibodies for oral cholera vaccine (OCV).

### Data analysis

Two independent reviewers (JAC and EPP) assessed eligibility of each full-text article; a third (AJP) arbitrated for cases without consensus. One reviewer (JAC) extracted data, and a second (EPP) validated data extraction for 10% of studies. Descriptive and quantitative data were entered into a spreadsheet based on the Cochrane data extraction tool.^21^ If more than one intervention or oral vaccine were reported in the same study, data were extracted separately for each. For studies with a factorial design, data were only extracted from the combined group if there was no interaction between interventions, otherwise data from individual groups were used. Where numerical data were not reported, we requested data from authors or used GetData Graph Digitiser to extract results from figures. We assessed quality of evidence for each study using Grading of Recommendations, Assessment, Development and Evaluation (GRADE) criteria^22^ (appendix p 9).

To assess the effect of each intervention on seroconversion, we did random-effects meta-analyses in the R package *metafor*.^23^ We calculated summary relative risk (RR) estimates and 95% CIs for each vaccine evaluated in two or more studies. If more than one vaccine was assessed for a given intervention, we did a pooled analysis combining across vaccines, and a mixed-effects analysis with vaccine type as a moderator. For mixed-effects models, we identified whether heterogeneity associated with vaccine type or residual heterogeneity was significant via χ^2^ tests.^24^ If residual heterogeneity was significant (p<0·05), we assessed age, setting, and background immunogenicity (seroconversion rate in the control group) as secondary moderators. We used funnel plots to check for publication bias and tested for asymmetry using Egger's test. Further details of the analysis are provided in the appendix (p 10). All analyses were done in R (version 3.4.1).

### Role of the funding source

The funder had no role in study design, data collection, data analysis, data interpretation, or writing of the report. The corresponding author had full access to all data in the study and had final responsibility for the decision to submit for publication.

## Results

**Figure 1:**
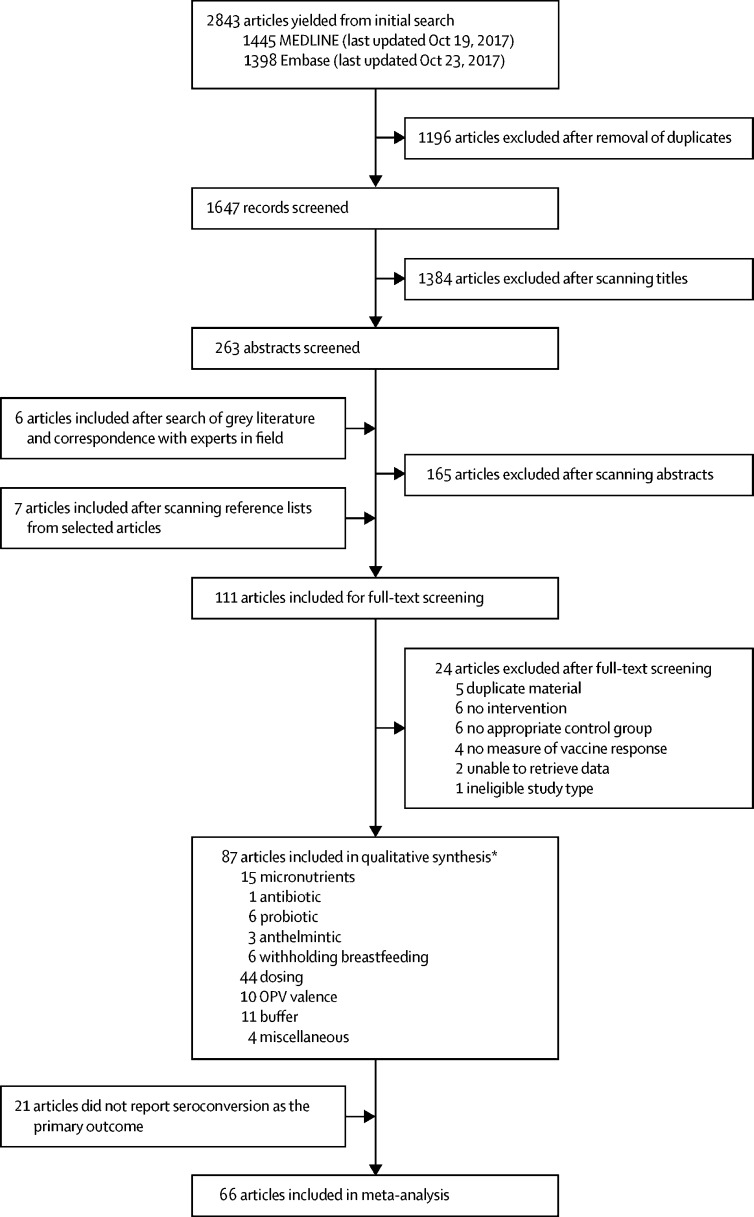
Study selection OPV=oral polio vaccine. *Studies exploring multiple interventions are duplicated within this breakdown.

Of 2843 articles, 87 were eligible for qualitative synthesis (figure 1). The studies are summarised in the table and the full dataset is available online. 74 (85·1%) were randomised trials and 13 (14·9%) non-randomised trials. The studies were done in 38 countries between 1972 and 2017 (appendix p 11) and enrolled between 30 and 225 998 participants (median 301, IQR 128–718). 11 studies (12·6%) were done in adults.

**Table:** Overview of 87 intervention studies included in the systematic review

		**Oral poliovirus vaccine**	**Oral rotavirus vaccine**	**Oral cholera vaccine**	**Oral typhoid vaccine**
Total studies (n)*		46	24	15	9
Intervention					
	Anthelmintic	0	0	2	1
	Antibiotic†	1	0	0	0
	Breastfeeding withheld	2	3	1	0
	Buffer	1	4	3	3
	Delayed first dose	0	4	0	0
	Early first dose†	1	0	0	0
	Extra dose or doses	1	6	1	2
	Extra dose at birth	5	1	0	0
	Miscellaneous†	3	0	1	0
	Narrow dose interval	3	1	0	1
	OPV valence	10	NA	NA	NA
	Other micronutrients†	2	0	0	1
	Probiotic	1	2	2	1
	RVV separated from OPV	7	7	NA	NA
	Vaccine inoculum	7	0	4	0
	Vitamin A	4	0	1	1
	Zinc	1	1	4	0
Age group					
	<1 month	19	1	0	0
	1–12 months	24	23	3	0
	1–15 years	2	0	4	6‡
	≥16 years	1	0	8	3
	Mean age (SD; months)	4·2 (7·9)	1·9 (1·3)	141·6 (163·3)	187·9 (133·8)
Sex					
	Men (%)	51·3	45·7	50·5	55·9
	Women (%)	48·7	54·3	49·5	44·1
Location					
	Africa	8	6	2	1
	Asia	25	10	7	2
	Europe	5	3	2	2
	Americas	8	4	4	4
	Oceania	0	1	0	0
Study size					
	<50	7	1	2	2
	50–500	37	21	13	2
	>500	2	2	0	5
Total seroconversion data (n)		8838	8954	1395	353 030

RVV=oral rotavirus vaccine. OPV=oral poliovirus vaccine.

* Of 87 unique studies, some studies examined two or more interventions and some reported on multiple oral vaccine targets (appendix p 12).

† There were insufficient studies (fewer than two) of antibiotics, early first dose, other micronutrients, and miscellaneous interventions (maternal vitamin A, horse anti-serum, soya formula, and *Escherichia coli* K-12) for inclusion in the meta-analysis.

‡ Most typhoid studies recruited children aged between 5 and 22 years.

22 interventions were assessed (14 adjunctive interventions and eight vaccine design or delivery interventions), grouped into 17 categories: anthelmintic therapy (n=3), antibiotic therapy (n=1), probiotic supplementation (n=6), zinc supplementation (n=6), vitamin A supplementation (n=6), other micronutrient supplementation (n=3), withholding breastfeeding (n=6), extra dose or doses (n=10), extra dose given at birth (n=6), early first dose (n=1), delayed first dose (n=4), shortened interval between doses (n=5), RVV given with versus without OPV (n=7), increased vaccine inoculum (n=11), different OPV valences (n=10), inclusion of buffer (n=11), and miscellaneous (n=4, appendix p 4). No studies of water, sanitation, or hygiene interventions were found. 12 studies included more than one intervention, of which two employed a factorial design (appendix p 12). OPV was the most common vaccine studied (46 studies, 52·9%), followed by RVV (n=24, 37·6%), OCV (n=15, 17·2%), and oral typhoid vaccine (n=9, 10·3%).

Of the 87 studies, 66 (from 13 intervention categories) were eligible for inclusion in the meta-analysis (table). Eight studies (9·3%) included in the qualitative synthesis reported vaccine efficacy as the primary outcome. Most studies (n=66, 75·9%) reported vaccine seroconversion as the primary outcome. Additional immunogenicity characteristics are described in the appendix (p 13).

GRADE scoring is reported in the full dataset. 11 studies (12·6%) had a low risk of bias across all domains of study quality, 76 (87·4%) had an unclear risk of bias in at least one domain, and 42 (48·3%) had a high risk of bias in two or more domains (appendix p 14). 30 studies were downgraded during GRADE assessment for indirectness, mostly because of poor generalisability (ie, not studying children or not done in an LMIC). Potential publication bias, assessed using funnel plots, was identified in seven intervention categories (appendix p 26).

**Figure 2:**
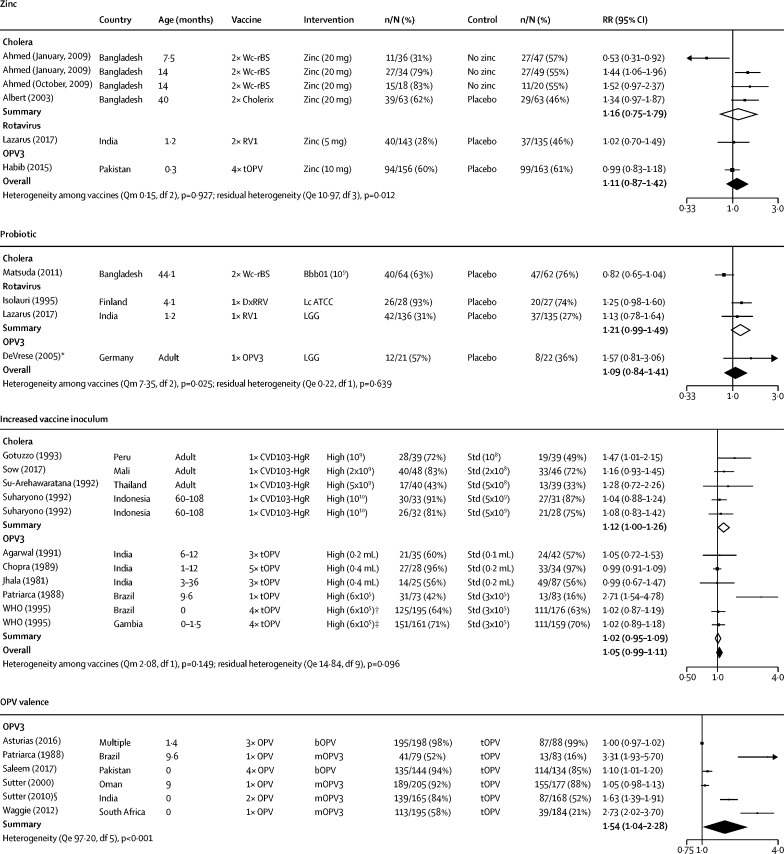
Effect of adjuncts or vaccine composition on seroconversion to oral vaccines Forest plot showing the effects of zinc supplementation, probiotics, increased vaccine inoculum, and OPV valence on seroconversion to OCV, RVV, or OPV. Bbb01=*Bifidobacterium breve* 01. Lc ATCC=*Lactobacillus casei* ATCC. LGG=lactobacillus GG. OPV=oral poliovirus vaccine. bOPV=bivalent OPV. mOPV=monovalent OPV. tOPV=trivalent OPV. RVV=rotavirus vaccine. RV1=Rotarix monovalent vaccine. Qm=Q statistic for moderator effect. *This study also examined another probiotic, *Lactobacillus casei*, with similar results. We excluded these data from the forest plot to avoid replication of the control group. †This study also included an arm comparing bOPV with tOPV. We excluded these data from the forest plot to avoid replication of the control group.

Six randomised controlled trials reported oral vaccine seroconversion following vitamin A supplementation. Among five studies eligible for meta-analysis (four on OPV, one on OCV), there was no significant effect of vitamin A supplementation on seroconversion (overall RR 1·01, 95% CI 0·99–1·03). There were six studies of zinc supplementation, of which five were included in the meta-analysis (three on OCV, one on RVV, and one on OPV). Overall, there was no significant effect of zinc supplementation on seroconversion (1·11, 95% CI 0·87–1·42; figure 2). A factorial trial in Bangladesh reported an interaction between vitamin A and zinc supplementation, whereby zinc combined with vitamin A increased seroconversion to OCV more than zinc alone.^25^ Single studies reported several other micronutrient interventions. There was no evidence that vitamin A given to mothers post partum,^26^ selenium capsules,^27^ or oral iodine^28^ had an effect on OPV immunogenicity, and giving multiple micronutrients had no effect on response to oral typhoid vaccine.^29^

Six studies assessed withholding breastfeeding for 1–10 h (mean 3·5, SD 3·4) around the time of oral vaccination; five were eligible for meta-analysis (one on OCV, three on RVV, one on OPV). There was no evidence of benefit for seroconversion to OCV, RVV, or OPV (overall RR 0·93, 95% CI 0·75–1·14; appendix p 15).

Six studies reported probiotic interventions, of which four were eligible for meta-analysis (one on OCV, two on RVV, one on OPV). In each study, daily probiotics were started 7 days before vaccination and continued for at least 3 weeks after; three studies used lactobacillus^30^, ^31^, ^32^and one used bifidobacterium.^33^ There was no evidence of an effect on seroconversion to OCV, RVV, or OPV (overall RR 1·09, 95% CI 0·84–1·41; figure 2).

32 studies reported changes in vaccine formulation (addition of a buffer, increased inoculum, or altered OPV valence); 28 were eligible for meta-analysis (eight on buffer, 11 on inoculum, nine on OPV valence). There was weak evidence that adding buffer increased seroconversion to OCV (RR 1·32, 95% CI 0·98–1·78), although there were few participants (n=219). Two typhoid studies showed increased vaccine efficacy when reconstituted in buffer compared with a capsule control.^34^, ^35^ Overall, when combining data for all vaccines, there was weak evidence of improved seroconversion with buffer (1·03, 95% CI 0·98–1·09; appendix p 16). Increases in vaccine inoculum showed some evidence of benefit for seroconversion across four studies for OCV (1·12, 1·00–1·26), but not for OPV (1·02, 0·95–1·09; figure 2). Nine studies exploring adjustments to OPV valence were included in the meta-analysis; seven used monovalent OPV and two used bivalent OPV. Compared with trivalent OPV, monovalent and bivalent OPV significantly increased seroconversion (1·51, 95% CI 1·20–1·91), with a consistent effect across OPV1 and OPV3 (appendix p 19).

**Figure 3:**
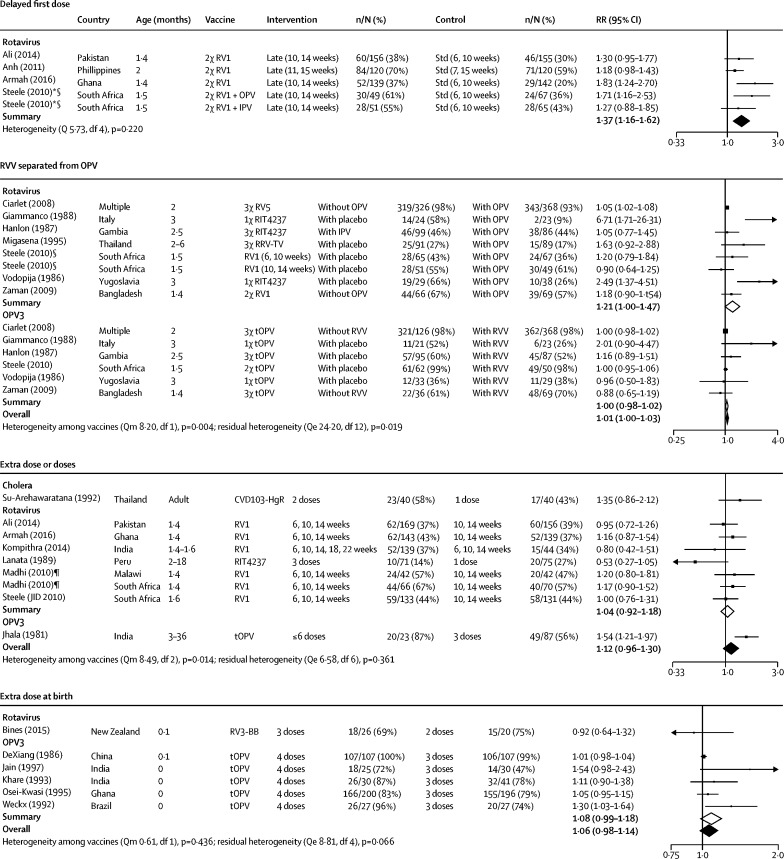
Effect of dosing on seroconversion to oral vaccines Forest plot showing the effects of delaying the first dose, separating RVV from OPV, and giving extra doses on seroconversion to OCV, OPV, or RVV. IPV=inactivated poliovirus vaccine. OPV=oral poliovirus vaccine. bOPV=bivalent OPV. mOPV=monovalent OPV. tOPV=trivalent OPV. RVV=rotavirus vaccine. RV1=Rotarix monovalent vaccine. RV5=RotaTeq pentavalent vaccine. Qe=Q statistic for residual heterogeneity. Qm=Q statistic for moderator effect. *Intervention and control group recruited separately. †Centrifuged. ‡Filtered. §Exact sample sizes were not reported for immunogenicity data and were therefore estimated by assuming that loss-to-follow-up rates reported in figure 1 of the trial report were evenly distributed across groups. ‡Study included both a 6-week plus 10-week dose schedule and a 10-week plus 14-week dose schedule. The 10-week plus 14-week schedule was selected as the control group to ensure consistency with other studies and to delineate the effect of extra doses from delayed doses (considered in a separate comparison). ¶Immunogenicity data extracted from Madhi et al^145^ and Cunliffe et al.^146^ Exact sample sizes were not reported for Malawi data; we therefore assumed that the 85 RVV recipients were distributed 1:1 across the 2-dose and 3-dose schedules (n=42 per group) and used the reported seroconversion rates (47·2% and 57·1%) to estimate the number of infants who seroconverted.

19 studies investigated changes to vaccine dosing; 14 were eligible for meta-analysis, assessing additional doses (n=8; one on OCV, six on RVV, one on OPV) or additional birth dosing (n=6; five on OPV, one on RVV). There was weak evidence that increasing the number of doses improved seroconversion (RR 1·12, 95% CI 0·96–1·30; figure 3). Two studies reporting RVV efficacy as the primary outcome also showed weak evidence of benefit in increasing from two to three doses.^36^, ^37^ There was weak evidence that an additional birth dose of OPV increased OPV3 seroconversion (RR 1·08, 95% CI 0·99–1·18), and findings remained similar in the combined meta-analysis, which included a rotavirus study^38^ (1·06, 95% CI 0·98–1·14; appendix p 17).

17 studies reported altered timing of vaccine administration (five narrowed dose interval, four delayed first dose, one early first dose, and seven staggered RVV and OPV administration); 15 were eligible for meta-analysis. We found no evidence that narrowing the dosing interval (from 4–8 weeks to 1–4 weeks, assessed in one study of RVV, two of OPV) benefited seroconversion (RR 0·98, 95% CI 0·94–1·02; appendix p 19), although these trials were primarily designed as non-inferiority studies. Conversely, delaying the first dose of RVV by 4 weeks (four studies) significantly increased seroconversion rates (1·37, 1·15–1·61; figure 3). We found no evidence that staggered administration of RVV and OPV affected OPV seroconversion (1·00, 0·98-1·02), with consistent findings across all OPV serotypes (appendix p 21). By contrast, there was some evidence that children given RVV alone or separated from OPV were more likely to seroconvert against rotavirus compared with children given RVV and OPV concomitantly (1·21, 95% CI 1·00–1·47; figure 3).

Three studies examined the effect of anthelmintics on oral vaccine responses^39^, ^40^, ^41^ with no overall effect on seroconversion to OCV (RR 1·26, 95% CI 0·63–2·53) or typhoid (appendix p 15). Only one antibiotic study was identified.^42^ This randomised controlled trial found no effect of oral azithromycin on OPV immunogenicity, although antibiotics reduced faecal biomarkers of environmental enteric dysfunction. There was no evidence that soy-based formulas,^43^ compared with human milk or conventional formula, affected OPV immunogenicity. One study of anti-human γ-globulin horse serum and another of inactivated *Escherichia coli* K12 showed a positive effect on OPV and OCV responses, respectively;^44^, ^45^ however, both were downgraded in GRADE assessment to a score of one or less (very low quality evidence) on the basis of study quality and indirectness.

Overall, there was considerable heterogeneity between studies. For three interventions (extra dose or doses, probiotics, and separating RVV from OPV), the intervention effect differed significantly according to vaccine type (appendix p 26); for four interventions (breastfeeding, OPV valence, separating RVV from OPV, and zinc), there was significant residual heterogeneity not explained by vaccine type (appendix p 26). For zinc, the effect on vaccine response was significantly greater for older than for younger children (appendix p 27). For OPV valence and separating RVV from OPV, background immunogenicity was negatively correlated with intervention effect (appendix p 27). Notably, the beneficial effect of staggered administration for RVV seroconversion was strongest in two studies with low background immunogenicity that administered only a single dose of RVV.^46^, ^47^ For withholding breastfeeding, neither age, setting, nor background immunogenicity were sig-nificant secondary moderators (appendix p 27).

Because of variation in timing of post-vaccine titre measurements, we did a sensitivity analysis, excluding 19 studies that measured seroconversion outside our prespecified windows (appendix p 8). The inferences for each intervention remained unaltered in meta-analysis (appendix p 28).

**Figure 4:**
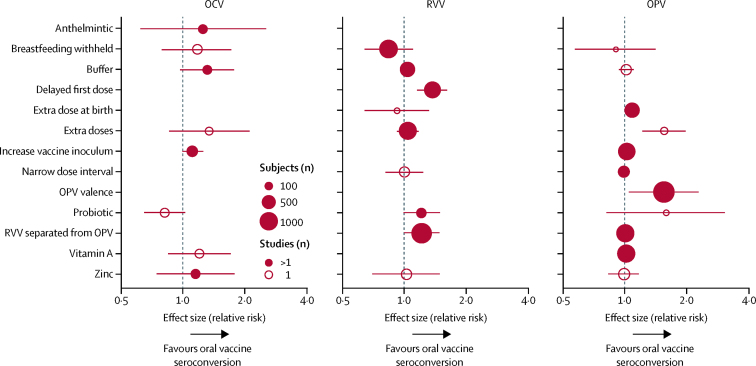
Meta-analysis summary Summary effect sizes from forest plots for each intervention according to vaccine type (OCV, RVV, and OPV). Error bars represent 95% CI and the size of the circles corresponds to the number of participants. OCV=oral cholera vaccine. OPV=oral poliovirus vaccine. RVV=rotavirus vaccine.

Summary effect sizes for each meta-analysis, grouped by vaccine, are shown in figure 4. Overall, RRs for seroconversion across all interventions ranged between 0·93 and 1·54. For OCV, there was weak evidence that all interventions (except probiotics) improved seroconversion. For RVV, delayed administration of the first dose was the most effective intervention; there was some evidence that staggered RVV and OPV and probiotics increased RVV seroconversion. For OPV, adjusting valence was the most effective intervention. Extra doses of OPV were also effective in one small study.^48^

## Discussion

Oral vaccine underperformance is a major public health problem in LMICs. In this systematic review, we identified 87 studies assessing interventions aimed at improving oral vaccine immunogenicity, done over the past four decades. Typically, there were few studies for a given intervention and effect sizes were small. Delayed RVV administration and altered OPV valence were the only effective approaches identified from the available evidence.

Our finding that delaying the first dose of RVV improves seroconversion is consistent with another systematic review.^19^ The mechanism for increased immunogenicity when RVV is delayed is probably a combination of less interference from maternal antibodies^49^ and maturation of infant immune function.^50^ Delayed RVV administration might also mitigate the inhibitory effect of OPV; for example, we found some evidence (driven primarily by studies involving a single RVV dose) that concurrent administration of OPV and RVV leads to lower seroconversion to RVV, consistent with in-vitro observations.^51^ The decision to delay administration of RVV on the basis of our findings should be weighed against the risks of postponing protection against early natural infection, which might increase the risk of severe disease. It would be useful to compare the frequency and severity of infections and cost-effectiveness between the current approach and a delayed strategy using modelling, to better inform scheduling decisions. Reducing OPV valence also showed clear evidence of benefit for seroconversion. Enhanced immunogenicity of monovalent or bivalent OPV compared with trivalent OPV reflects the inhibitory effect of Sabin 2 vaccine virus on the uptake of other serotypes. This finding suggests that the switch from trivalent OPV to bivalent OPV (which does not contain Sabin 2 virus) in 2016—motivated primarily by the need to mitigate the risk of vaccine-derived poliovirus outbreaks^52^—has probably improved immunity to seretypes 1 and 3.

Several interventions showed weak evidence of benefit, but effect sizes were generally small and the lower bound of the 95% CI did not exclude unity. There was weak evidence of increased seroconversion with oral vaccine buffering, which is designed to protect key components from destruction by gastric acid. However, administering oral vaccines that incorporate sufficient buffer can be challenging in young children, and there might be a reduced effect in this age group because they have little gastric acid.

We found some evidence that increasing vaccine inoculum improved seroconversion to OCV, but not to OPV. For OCV, increasing the inoculum from 1 × 10^8^ to 1 × 10^9^ colony-forming units (CFUs) modestly improved seroconversion rates (RRs of 1·16–1·47), while further increases (from 5 × 10^9^ to 1 × 10^10^ CFUs) did not confer any additional benefit.^53^ Together, these findings suggest that OCV immunogenicity might plateau as the administered dose increases. OPV already contains more than 1 × 10^5^ median cell-culture infectious doses of each Sabin poliovirus serotype, and any further increases in the inoculum might have limited effect.

There was some evidence that additional oral vaccine doses were beneficial for OPV in the single eligible study. This is consistent with the observation from supplementary immunisation programmes that additional doses are immunogenic and that efficacy increases with a constant per-dose response rate.^48^, ^54^ For RVV, we observed no significant benefit of giving additional Rotarix vaccine (3–5 doses *vs* 1–3 doses) up until age 22 weeks. However, RVV studies typically compared a schedule of RVV given at age 6, 10, and 14 weeks with RVV given at age 10 weeks and 14 weeks (rather than the standard schedule given at age 6 weeks and 10 weeks for Rotarix), which might explain the lack of benefit observed. A booster dose of RVV at age 9 months induced IgA seroconversion in more than 40% of Bangladeshi infants who had received two previous doses of vaccine,^55^ highlighting the potential benefit of additional doses in later infancy.

Finally, our meta-analysis showed that a birth dose of OPV had no overall effect on seroconversion rates. However, these data do not capture the benefits of vaccinating at birth to improve coverage.^56^ Moreover, a benefit of vaccination in the neonatal period is protection from early natural infection, which occurs frequently in low-income countries.^57^

For most interventions, we found no evidence of effect on oral vaccine performance. In the meta-analysis, there was no benefit of vitamin A supplementation for OPV or OCV immunogenicity in Asia or Africa. We also found no overall effect of zinc supplementation on oral vaccine responses across five studies, despite its role in mucosal protection.^58^ However, secondary analyses showed that zinc supplementation had a greater effect on oral vaccine responses among older children. Vitamin A and zinc deficiency vary with age and setting, and true deficiency might have been under-represented in the included trials. Three randomised trials showed no effect of temporarily withholding breastfeeding on RVV, live-attenuated OCV (CVD 103-HgR), or OPV immunogenicity. In one trial in Pakistan, there was, paradoxically, weak evidence for increased RVV seroconversion rates in the group with liberalised breastfeeding.^59^ Notably, breastfeeding was only withheld for short periods (mean 3·2 h); however, the feasibility and value of withholding breastfeeding for longer is doubtful. Instead, other novel methods such as vector systems should be pursued to tackle maternal antibody interference.^60^ The results from our meta-analysis show no significant benefits overall for probiotics, despite findings from individual studies that probiotics can increase levels of specific antibodies to oral vaccines. This finding might be due to the heterogeneity of probiotic strains, reflecting an incomplete understanding of which are most likely to confer benefits. In a previous systematic review of the effects of probiotics on oral and parenteral vaccines, a total of 40 different probiotics were tested, with variations in dose, purity, and timing of administration.^18^ The review concluded that the potential benefit of probiotics was strongest for oral vaccines, although only two studies were done among children.

Our review highlights several interventions that merit further study. First, although we found no overall benefit from increasing the number of RVV doses, additional doses given at birth or later in infancy may have a greater impact, particularly as WHO guidelines now allow for later dosing of RVV.^61^ There is also interest in early oral vaccination using neonatal strains of rotavirus. One candidate (RV3-BB) was shown to be efficacious against severe gastroenteritis in Indonesian infants.^62^

Second, of the six probiotic studies included in the qualitative analysis, four were done in European countries and three of these recruited adult participants. It is therefore difficult to draw conclusions about the value of probiotics in improving oral vaccine efficacy among infants in LMICs. Moreover, the association between bacterial microbiota composition and oral vaccine responses remains equivocal.^63^ Further studies might help to better define microbiota-directed therapies that will benefit vaccine efficacy.

Third, we did not find any studies exploring the effects of water, sanitation, or hygiene on oral vaccine responses, although one study is underway in Zimbabwe.^64^ Fourth, no studies investigated adjustments to RVV inoculum (with the exception of early pre-licensure immunogenicity dose-finding trials, which were excluded).^65^, ^66^ Our search of the grey literature highlighted one trial, underway in Bangladesh, exploring the effects of an increased inoculum on RVV immunogenicity (NCT02992197). Finally, only one antenatal intervention trial was identified in this systematic review.^26^ In this study, OPV seroconversion was similar among infants of mothers randomised to receive vitamin A or placebo in pregnancy. The antenatal period might provide a window of opportunity to assess the effect of maternal interventions such as macronutrients, micronutrients, probiotics, or antibiotics, since oral vaccine underperformance occurs so early in infancy.

Our study has several limitations. First, we used seroconversion as our primary outcome for meta-analysis; however, serological markers do not always correlate with protection from disease, particularly for RVV, for which rotavirus-specific IgA is a poor correlate of protection in low-income countries.^67^ Second, there were differences in study design and significant heterogeneity between studies for several interventions, despite our strict inclusion criteria, probably reflecting the range of ages, settings, and vaccine types across studies. However, we undertook meta-regression to provide insights into factors that drive heterogeneity and did sensitivity analyses restricted to studies that measured vaccine immunogenicity during a narrow time window, which did not change our inferences. Third, several studies reported high seroconversion rates in intervention and control groups, which might have obscured detection of a beneficial treatment effect. For example, effect size was negatively correlated with background immunogenicity for valence and separation of RVV from OPV. Studies with high background immunogenicity might also have contributed to the potential publication bias observed for several interventions because their small effect sizes and standard errors might create an apparent skew in funnel plots and Egger's test p values. Finally, comparisons often included small numbers of studies, subdivided by vaccine type, which were sometimes done among adults in high-income countries. Therefore, the available evidence was sometimes insufficient to determine the generalisability of interventions for the most relevant populations of interest (mostly infants in LMICs), which is apparent from the substantial proportion of studies that were downgraded for indirectness in the GRADE assessment.

Oral vaccines provide tremendous benefits in developing countries; however, they are failing to reach their full potential. Very few strategies substantially improve oral vaccine immunogenicity, and those that do have modest effects. There is a need to better understand the causes of oral vaccine failure to inform more effective interventions; however, overcoming the multiple factors that probably underlie oral vaccine failure in LMICs might be difficult.^3^ In addition to optimising the effectiveness of intervention approaches, other factors such as safety, feasibility, and affordability should be considered. Immunisation strategies in LMICs must therefore also consider alternatives to oral vaccines. Bypassing the gut through parenteral administration, for example, is an alternative strategy not evaluated in this review. In 2018, a Vi-polysaccharide tetanus-toxoid conjugate typhoid vaccine received WHO pre-qualification, supported by field estimates showing long-term protection,^68^ and a parenteral rotavirus vaccine has been shown to be immunogenic in South African infants.^69^ Another strategy being explored is the use of mucosal adjuvants such as dmLT, a detoxified version of *Escherichia coli* enterotoxin, combined with inactivated poliovirus vaccine.^70^ However, whether these interventions can be scaled up effectively is unclear. Meanwhile, introducing changes in oral vaccine scheduling, such as deferred RVV dosing, after a careful assessment of the costs and benefits, could improve the effect of oral vaccines for children in LMICs countries and reduce the global burden of diarrhoeal disease.

## Data sharing

Extracted data for all included studies are available online. The analysis code is available. All figures and statistical outputs are available in online.
