# Supplementary material for: Interventions to improve oral vaccine performance: a systematic review and meta-analysis
Source: Lancet Infect Dis. 2019 Feb;19(2):203–14. doi: 10.1016/S1473-3099(18)30602-9 (PMC6353819; doi:10.1016/S1473-3099(18)30602-9)
Supplement: Supplementary appendix [file mmc1.pdf]

THE LANCET Infectious Diseases

Supplementary webappendix

This webappendix formed part of the original submission and has been peer reviewed. We post it as supplied by the authors.

Supplement to: Church J A, Parker E P, Kirkpatrick B D, et al. Interventions to improve oral vaccine performance: a systematic review and meta-analysis. *Lancet Infect Dis* 2019; **19:** 203–14.

Interventions to improve oral vaccine performance in developing countries: a systematic review and meta-analysis

James A Church MBBS [1,2], Edward P Parker PhD [3], Beth D Kirkpatrick MD [4], Nicholas C Grassly PhD [3], Andrew J Prendergast DPhil [1,2]

Table of Contents for Appendix

Supplementary methods		Page
i.	Rationale for intervention categories	2
ii.	Complete search strategy for Ovid Medline	5
iii.	List of experts in the field	7
iv.	Table S1: Preferred measures of immunogenicity	8
v.	Assessment of evidence quality	9
vi.	Table S2: GRADE scoring system	9
vii.	Additional methods	10
viii.	R markdown code	10
**Supplementary results**		
i.	Figure S1: Map depicting studies of interventions to improve OVs	11
ii.	Table S3: Trials testing multiple interventions	12
iii.	Immunogenicity characteristics	13
iv.	Figure S3: Risk of bias summary for all studies	14
v.	Forest plots and funnel plots for all interventions	15
a.	Footnotes	24
b.	Acronyms	25
vi.	Table S4: Summary of secondary analyses for each intervention	26
vii.	Table S5: Meta-regression secondary moderators	27
viii.	Sensitivity analysis	28
ix.	Table 1: Overview of studies (fully referenced version)	29
**Supplementary references**		30

Supplementary methods

i. Rationale for intervention categories

We compiled a list of both plausible and recognised interventions based on factors considered to play a role in oral vaccine underperformance. Much of this scientific rationale was based upon a detailed review by one of the authors considering the causes of impaired oral vaccine efficacy in developing countries[1]. Below we list the rationale for each of these intervention categories.

a. Micronutrients

Vitamin A encompasses a group of retinoid compounds (biological activity all-*trans*-retinol) which play an essential role in a number of physiological functions including immunity. Vitamin A is essential for healthy immune responses at mucosal surfaces and deficiency results in increased mortality and morbidity from measles, diarrhoea, blindness and anaemia[2]. Vitamin A deficiency is prevalent in regions where oral vaccines underperform and Vitamin A supplementation (VAS) is widely accepted and considered to be among the most important tools to reduce childhood mortality in children aged 6-59 months[3]. Animal studies have shown that Vitamin A deficiency impairs vaccine-elicited gastrointestinal immunity and that replacement with Vitamin A or its metabolite retinoic acid fully restores the mucosal immune response[4,5]. Vitamin A derivatives have also shown adjuvant potential in humans when given alongside oral and parenteral vaccines[6,7]. Zinc is an essential mineral involved in multiple aspects of cellular metabolism. Deficiency in zinc leads to growth retardation, loss of appetite and impaired immune function and is strongly correlated with increased diarrhoeal morbidity and mortality[8]. Several studies describe clear benefits of both supplemental and therapeutic zinc in protecting children from diarrhoeal disease[9]. Like Vitamin A, zinc deficiency is more prevalent in regions where oral vaccines underperform. Given its integral role in gut health both through intestinal epithelial repair and regulation of mucosal immune responses, it is plausible that deficiency may attenuate seroconversion to oral vaccines and that supplementation may improve immune responses to oral vaccines.

b. Antibiotics

Many children in developing countries have frequent, early and recurrent exposure to enteric pathogens from early life[10], which could impair the efficacy of an oral vaccine in several ways. First, enteropathogen exposure can cause diarrhoea, which reduces intestinal transit time, thereby lessening vaccine exposure. Second, enteropathogen exposure can accentuate mucosal innate immune responses, thereby impairing vaccine replication. For example, in children infected with non-polio enteroviruses or having diarrhoea at the time of vaccination, immunogenicity to oral poliovirus vaccine is significantly reduced[11]. Third, induction of innate and adaptive immune responses at the intestinal mucosa can cause perturbations to the gut microbiota[12], which might in turn interfere with oral vaccine responses (see *Probiotics* below). Fourth, pathogens scavenge and compete for energy sources which may interfere with the action and replication of live vaccine virus. Finally, repeated exposure to intestinal pathogens can contribute to chronic alterations in gut structure and function, characterised by increased permeability, reduced absorptive capacity and chronic inflammation, which together have been termed environmental enteric dysfunction (EED)[13]. Biomarkers of EED have been associated with reduced immune responses to oral poliovirus and rotavirus vaccines in some studies[14,15], though not in others. Given the potentially deleterious effect of enteric infection or colonisation on the mucosal immune response, a course of antibiotic therapy given around the time of vaccine administration may reduce enteropathogen carriage and improve oral vaccine performance.

c. Anthelminthics

Helminth infestation is prevalent among children in developing countries[16] and their geographical distribution has extensive overlap with areas in which oral vaccines underperform. Intestinal helminth infection is associated with substantial childhood morbidity including anaemia, malabsorption and stunting[17], as well as altered immune function[18]. As a result, geohelminths inhabiting the small intestine may interfere with the uptake of oral vaccines in the intestinal lumen. Anti-helminthics, a group of anti-parasitic drugs, are recommended by the WHO for periodic deworming to reduce morbidity among children living in endemic areas[19]. Treating helminth infections may enhance immune responses to oral vaccines. However, helminth infestation is rare in early infancy when routine oral vaccines are administered, so the benefits of this intervention may be limited to vaccines administered to older children.

d. Probiotics or prebiotics

The role of the intestinal microbiota on health and immunity is garnering increasing interest. Experiments in germ-free animal models have helped explain mechanisms by which the microbiota influences early immune development and responses[20]. In humans, a recent study described differences in the microbiota composition, including a decreased abundance of Batceroidetes, among Ghanaian infants who failed to respond to oral rotavirus vaccine[21]. Moreover, the microbiota of the Ghanaian infants who responded to oral rotavirus vaccine was more similar than non-responders to that of rotavirus-unvaccinated Dutch infants of matched age. However, a study in India found no significant association between microbiota composition and rotavirus vaccine immunogenicity[22]. Despite these conflicting data, it remains plausible that alterations to the intestinal microbiota can modulate response to oral vaccines. Probiotics are live microorganisms intended to have health benefits, which have been linked to actions that may directly or indirectly influence immune function. In principle, they have the capacity to alter the composition of the gut microbiota and communicate with many cell types, thereby enhancing barrier function, increasing mucin production and promoting IgA secretion. The same is true to a lesser extent with prebiotics, which are non-digestible fibre compounds designed to stimulate the growth and activity of advantageous commensal bacteria in the gut. As a result, well-chosen probiotics or prebiotics, or synbiotics (a combination of prebiotics and probiotics) may modify the intestinal environment in favour of robust mucosal responses to oral vaccines.

e. Withholding breastfeeding

It has been postulated that breastfeeding may attenuate immune responses to oral vaccines. Breast milk contains secretory IgA antibodies as well as innate immune factors such as lactoferrin which can inhibit the replication of live viruses[23]. There are also geographical differences in the composition of breast milk. Rotavirus neutralising titres in breast milk are higher in Bangladeshi mothers compared to mothers from the U.S.A[24], mirroring the geographical patterns of oral vaccine underperformance. It is therefore possible that withholding breastfeeding around the time of administration of an oral vaccine may enhance the mucosal immune response.

f. Dosing or schedule changes

The endgame to eradicate poliomyelitis has been challenged by oral vaccine underperformance and exemplifies strategies used to close immunity gaps. In some areas, despite high coverage and intensive use of OPV, polio eradication has remained challenging. There are probably several contributing factors (listed above) including a high force of infection. One approach to addressing these polio ‘hotspots’ has been to use higher potency vaccines and supplemental doses. In Uttar Pradesh, India, high potency mOPV1 and supplemental IPV has been shown to enhance OPV-induced mucosal immunity[25]. More recently, with the global elimination of Sabin 2, strategies have involved adjustments to the valence of the oral vaccine with bivalent and monovalent preparations being used in place of the traditional trivalent OPV.

Research tackling the underperformance of rotavirus vaccines has also explored dose adjustments (delayed dosing and or increased number of doses)[26-28]. Rotavirus vaccine is currently recommended at 6 and 10 weeks of age; however, in developing countries, doses at younger ages generally yield lower rotavirus vaccine responses. A post-hoc exploratory analysis of vaccine trial data showed that African children receiving the first dose of pentavalent rotavirus vaccine at <8 weeks had lower efficacy (23.7%; 95%CI: -8.2, 46.3) than those vaccinated at >8 weeks (59.1%; 95%CI: 34.0, 74.6)[29]. Reasons for this may include the interference of concomitantly administered OPV and maternally acquired antibodies. IgA seroconversion was reduced among participants with higher levels of pre-vaccination maternally-derived IgG[26,30]. A delayed or additional dose of rotavirus vaccine, given after 10 weeks, may limit interference from circulating maternal antibodies and live oral polio vaccine virus as well as benefiting from a more mature infant immune system. Additional rotavirus vaccine doses however must be weighed up against the increased risk of intussusception when rotavirus vaccine is given later in childhood.

g. WASH (water, sanitation & hygiene) interventions

Safe drinking water, access to sanitation and hygiene have long been viewed as key determinants of population health. The term WASH captures several interventions, including access to and treatment of drinking water, safe disposal of faeces (including infant and animal faeces) and hand-washing with soap. In many developing countries, children grow up in conditions of poor WASH. It is possible that this leads to increased subclinical carriage of enteric pathogens, diarrhoea and EED, altering the intestinal environment and reducing

immunogenicity of oral vaccines. If this hypothesis is correct, it is logical that interventions to improve WASH may prevent pathogen carriage, diarrhoea and EED and thereby enhance responses to oral vaccines.

h. Other plausible interventions

Given the numerous factors that have been linked to the underperformance of oral vaccines, it is possible that other interventions have been tested outside of those listed above. One example would be nutritional interventions beyond zinc and Vitamin A supplementation. Malnutrition underlies 45% of deaths in children under 5 years in developing countries and there is high degree of overlap between regions affected by malnutrition and oral vaccine failure[31]. Although data regarding the association between nutritional status and oral vaccine response are conflicting[1], it is possible that other interventions to improve nutritional status in undernourished children may also improve responses to oral vaccines. For this reason, macronutrients as well as micronutrients have been included in our list of interventions.

Another potential window of opportunity is prenatal interventions. The first 1000 days (from conception to a child's second birthday) is increasingly recognized as a critical period of child growth and development, including dynamic intestinal adaptation and immune ontogeny. Environmental factors and maternal health from early pregnancy can also shape epigenetic changes in the developing fetus[32] and impact on later health and immunity. There is evidence, for example, that prenatal exposure to maternal helminth infections may modulate infant responses to vaccination and infectious pathogens[33,34]. It is therefore conceivable that a maternal anthelminthic intervention could boost immune responses to infant oral vaccines. Results of a large randomised controlled trial have shown that neither albendazole nor praziquantel given during pregnancy affect infant immune responses to BCG, tetanus and measles immunisations[35]. In this trial, oral vaccine responses were not examined; however, another study in Ecuador evaluated oral vaccine responses in the context of maternal helminth infection in pregnancy and paradoxically showed a protective effect, with maternal infection associated with higher infant IgA titres to oral polio and rotavirus vaccine antigens[36].

ii. Complete search strategy for Ovid Medline

The Ovid Medline search was first conducted on the 30th March 2017 and then refreshed on the 23rd October 2017.

#	Searches
1	exp Vaccination/
2	Vaccin*.mp.
3	1 or 2
4	Poliovirus/
5	Poliovirus:.mp.
6	Polio.mp.
7	Rotavirus/
8	Rotavirus:.mp.
9	Cholera/
10	Cholera:.mp.
11	Typhoid Fever/
12	Typhoid:.mp.
13	Salmonella typhi/
14	Salmonella typhi:.mp.
15	Shigella/
16	Shigella:.mp.
17	4 or 5 or 6 or 7 or 8 or 9 or 10 or 11 or 12 or 13 or 14 or 15 or 16
18	3 and 17
19	Poliovirus Vaccine, Oral/
20	Poliovirus Vaccines/
21	Rotavirus Vaccines/
22	Cholera Vaccines/
23	Typhoid-Paratyphoid Vaccines/
24	Shigella Vaccines/
25	19 or 20 or 21 or 22 or 23 or 24
26	18 or 25
27	Immunogenicity.mp.
28	Immunogenicity, Vaccine/
29	Response.mp.
30	Seroresponse.mp.
31	Seroconversion.mp.
32	Shedding.mp.
33	Virus shedding/
34	Efficacy.mp.
35	Titre.mp.
36	Titer.mp.
37	Antibodies, Viral/
38	Antibodies, Bacterial/
39	Performance.mp.
40	27 or 28 or 29 or 30 or 31 or 32 or 33 or 34 or 37 or 38 or 39
41	26 and 40
42	Zinc.mp.
43	**41 and 42**
44	Vitamin A.mp.
45	**41 and 44**
46	Micronutrient.mp.
47	Micronutrients/
48	46 or 47
49	**41 and 48**
50	Macronutrient.mp.
51	**41 and 50**
52	Anti-bacterial agents.sh.
53	Antibiotic.mp.
54	52 or 53

55	**41 and 54**
56	Anthelmintics.sh.
57	Anthelmintic.mp.
58	Albendazole.mp.
59	Praziquantel.mp.
60	56 or 57 or 58 or 59
61	**41 and 60**
62	Prebiotic.mp.
63	Probiotics.sh.
64	Probiotic.mp.
65	LGG.mp.
66	62 or 63 or 64 or 65
67	**41 and 66**
68	Breast Feeding.sh.
69	Breastfeed:.mp.
70	68 or 69
71	**41 and 70**
72	Dosing.mp.
73	Schedule.mp.
74	Ad.fs.
75	72 or 73 or 74
76	**41 and 75**
77	limit 76 to (clinical trial, all or meta analysis or multicenter study or observational study or systematic reviews)
78	Buffer.mp.
79	**41 and 78**
80	(hand*1 adj3 (wash* or clean* or disinfect*)).mp.
81	(hand*1 adj3 hygien*).mp.
82	Hand washing.sh.
83	(handwashing or hand washing).mp.
84	Hygiene/
85	(hygiene adj2 educat*).mp.
86	Sanita*.mp.
87	Water Supply/
88	Water Purification/
89	(Soaps/ or soap.mp.) adj3 (water* or hygien* or educat* or wash*).mp.
90	Sanitation/
91	(latrine*1 or toilet*1 or water closet*1 or privy).mp.
92	80 or 81 or 82 or 83 or 84 or 85 or 86 or 87 or 88 or 89 or 90 or 91
93	**41 and 92**

iii. List of experts in the field contacted

- Joe Brown (Georgia Tech); joe.brown@ce.gatech.edu
- Roma Chilengi (Centre for Infectious Disease Research in Zambia); Roma.Chilengi@cidrz.org
- Oliver Cumming (London School of Hygiene & Tropical Medicine); Oliver.Cumming@lshtm.ac.uk
- Roger Glass (Fogarty International Center); rglass@emory.edu
- Paul Kelly (Queen Mary University of London); m.p.kelly@qmul.ac.uk
- Margaret Kosek (Johns Hopkins University); mkosek@jhu.edu
- Bill Petri (University of Virginia); wap3g@virginia.eduv
- Duncan Steele (Bill & Melinda Gates Foundation); duncan.steele@gatesfoundation.org
- Anita Zaidi (Bill & Melinda Gates Foundation); Anita.zaidi@aku.edu
- Gagandeep Kang (Christian Medical College, India); gkang@cmcvellore.ac.in
- Beate Kampmann (Imperial College London & MRC Gambia); b.kampmann@imperial.ac.uk
- Nigel Cunliffe (University of Liverpool); N.A.Cunliffe@liverpool.ac.uk

iv. **Table S1:** Preferred measures of immunogenicity for different oral vaccine types.

**Vaccine**	**Measures of immunogenicity**		
	**Preferred measure**[1] (definition)	**Timing**	**Alternative measure**
Oral poliovirus vaccine	Seroconversion (serum neutralizing AB titres ≥ 1:8 post vaccine)	21-28 days post last dose	Serum neutralizing AB titres<br>Polio virus shedding (‘take’)[2]<br>Anti-polio specific IgA or IgG (serum or stool)
Oral rotavirus vaccine	Seroconversion (≥3-fold rise or ≥20U/ml (if pre-vaccine titre <20U/mL) serum RV IgA AB titres post vaccine)	14-28 days post last dose	Serum RV IgA titres<br>RV complement-fixing AB<br>RV-sIgA or Copro IgA<br>Serum NAs[3] to different serotypes
Oral cholera vaccine	Seroconversion (≥4-fold rise serum vibriocidal AB titres post vaccine)	7-14 days post dose	Vibriocidal AB titres<br>Vibriocidal sIgA<br>Cholera LPS IgA or IgG<br>CTB IgA or IgG
Oral typhoid vaccine	Seroconversion (≥4-fold rise serum anti-LPS IgG or IgA AB post vaccine)	7-14 days post dose	Serum anti-LPS IgG or IgG<br>Intestinal sIgA<br>IgG and IgA ASC

[1] Deviations from these definitions were permitted provided that they did not breach our inclusion/exclusion criteria

[2] OPV shedding had to be measured between 1 and 4 weeks after vaccination

[3] NA = neutralising antibody, sIgA = secretory IgA, LPS = lipopolysaccharide, CTB = cholera toxin subunit B, ASC = antibody secreting cell

v. Assessment of evidence quality

The quality of evidence was assessed for each study using GRADE criteria, which evaluates type of evidence, risk of bias, consistency with other studies, directness to the research question and effect size.

Since observational studies were excluded from this review, all included studies were given an initial score of +4 for type of evidence. Risk of bias was assessed based on several quality areas including the allocation process, blinding, follow-up, withdrawal rate and sparse data. A problem with one of these factors incurred one negative point, a problem with two elements incurred two negative points up to a maximum of three negative points. Sub-studies nested within trials incurred a negative point if selection of subjects was not random and pre-specified. Where details of selection, allocation, blinding or other quality parameters were not detailed, the risk of bias was graded as ‘unclear’. If multiple quality fields were unclear, the study lost one point per two unclear quality fields. Studies also incurred a negative point if there were losses to follow-up or withdrawals in excess of 15%. Similarly, studies including older subjects or in high-income countries lost up to two points, due to indirectness to the primary research question. The final GRADE score was derived from the sum of all five categories of evidence and classified as high (≥4 points overall), moderate (3 points), low (2 points), or very-low quality evidence (one or less). We describe full details of this scoring system in Table S2.

vi. **Table S2:** The GRADE scoring system used for clinical evidence reviews, adapted from advice and resources prepared by the GRADE Working Group[37]

**Type of evidence**		
**Based on**	Study design	
**Initial score**	+4	RCTs/ SR of RCTs, +/– other types of evidence
	+2	Observational evidence (e.g., cohort, case-control)
Quality		
**Based on**	Blinding and allocation process	
	Follow-up and withdrawals	
	Sparse data	
	Other methodological concerns (e.g., incomplete reporting, subjective outcomes)	
**Score**	0	No problems
	–1	Problem with 1 element
	–2	Problem with 2 elements
	–3	Problem with 3 or more elements
**Inconsistency**		
**Based on**	Degree of consistency of effect between or within studies	
**Score**	+1	Evidence of dose response across or within studies (or inconsistency across studies is explained by a dose response); also 1 point added if adjustment for confounders would have increased the effect size
	0	All/most studies show similar results
	–1	Lack of agreement between studies (e.g., statistical heterogeneity between RCTs, conflicting results)
**Indirectness**		
**Based on**	The generalisability of population and outcomes from each study to our population of interest	
**Score**	0	Population and outcomes broadly generalisable
	–1	Problem with 1 element
	–2	Problem with 2 or more elements
**Effect size**		
**Based on**	The reported OR/RR/HR for comparison	
**Score**	0	Not all effect sizes >2 or <0.5 and significant; or if OR/RR/HR not significant
	+1	Effect size >2 or <0.5 for all studies/meta-analyses included in comparison and significant
	+2	Effect size >5 or <0.2 for all studies/meta-analyses included in comparison and significant

vii. Additional methods

a. Rationale for OPV outcome

For OPV, we only included seroconversion to poliovirus serotype 3 in the main meta-analysis. Rates of seroconversion are typically lowest for this serotype, giving more power to detect an effect of the interventions. Serotype-specific analyses, with study included as a random effect, can be found in the e-appendix (https://eparker12.github.io/oral_vaccine_interventions_metaanalysis_2018/). Study was also included as a random effect for pooled analyses of concomitant versus separate administration of RVV and OPV, wherein outcomes for both vaccines were reported for the same infants.

b. Rationale for choice of intervention study group

In studies where multiple permutations of an intervention were tested against a single control group, the intervention that differed most in its characteristics from the control was selected, in order to avoid replication of control group participants. For example, if a dose interval was shortened from 8 weeks in the control group to either 1 or 3 weeks in the intervention arms, the 1-week interval was selected. Finally, for studies reporting stratified outcomes (e.g. by age group), we treated the strata as separate studies.

viii. R markdown code

The full markdown code used for the statistical analyses is available online at the following Github repository:

https://github.com/eparker12/oral_vaccine_interventions_metaanalysis_2018

Supplementary results

i. **Figure S1**. Map depicting studies of interventions to improve oral vaccine performance. Colour denotes the oral vaccine studied; shape denotes the intervention evaluated; and size of the shape denotes the number of participants in the study.

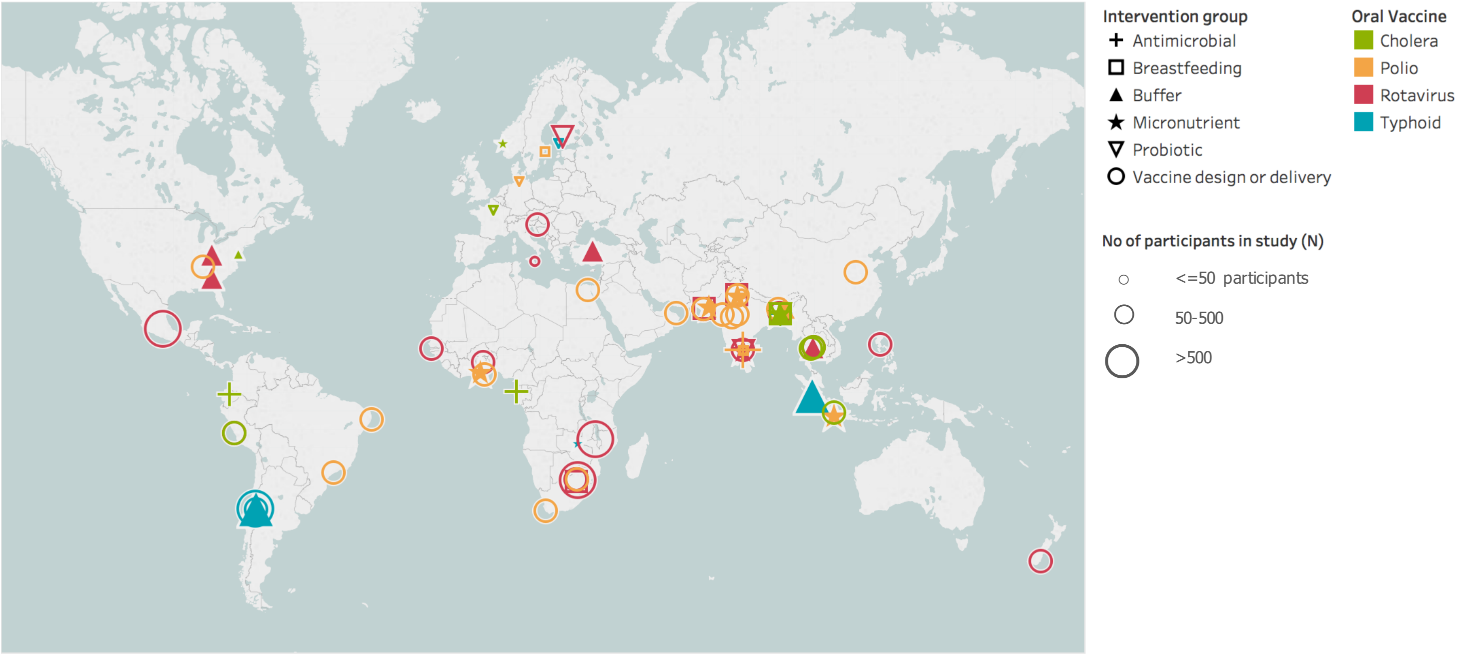

ii. **Table S3:** Trials testing multiple interventions.

Reference	Interventions (N, type)	Factorial design (Y/N)	Interaction (Y/N)	Combined arm data included (Y/N)
Ahmed (Vaccine 2009)[38]	3 (Zinc, Withholding breastfeeding, Buffer)	No	N/A	N/A
Albert (JID 2003)[39]	2 (Vitamin A, Zinc)	Yes	Yes (additive effect)	No
Ali (JID 2014)[26]	2 (Dose timing, dose number)	No	N/A	N/A
Anh (Vaccine 2011)[40]	2 (Dose timing, dose interval)	No	N/A	N/A
Armah (JID 2016)[27]	2 (Dose timing, dose number)	No	N/A	N/A
Lazarus (Vaccine 2018)[41]	2 (Zinc, Probiotic)	Yes	No	Yes
Steele (Vaccine 2010)[42]	2 (Dose timing, OPV/RV)	No	N/A	N/A
Jhala (Ind Ped 1981)[43]	2 (Vaccine inoculum, dose number)	No	N/A	N/A
Su-Arehawaratana (JID 1992)[44]	2 (Vaccine inoculum, dose number)	No (4 separate studies)	N/A	N/A
John (Vaccine 2011)[45]	2 (Vaccine inoculum, valency)	No (2 separate trials)	N/A	N/A
Patriarca (Lancet 1988)[46]	2 (Vaccine inoculum, valency)	No	N/A	N/A
Levine (Lancet 1987)[47]	2 (Buffer, dose interval)	No	N/A	N/A

iii. Immunogenicity characteristics

There was variability in the definitions used for seroconversion [summarised at osf.io/bemw6], in particular the interval between the final dose of vaccine and post-vaccine titre: 14-60 (mean 31·4) days for RVV, 21-270 (mean 42·0) days for OPV, 7-28 days (mean 13·6) days for OCV and 7-30 (mean 15·5) days for typhoid.

Of the studies also reporting pre- and post-vaccine antibody titres, the ratios of titres between study arms were highly comparable with seroconversion ratios (see Figure S2 below). Only one study reported a significant effect of the intervention on GMT without finding an impact on seroconversion[48].

Figure S2: Graph showing relationship between seroconversion ratio and post-vaccine titre ratio (intervention versus control) in studies reporting both outcomes.

Pearson r=0.55 (95% CI 0.36-0.70), p<0.0001

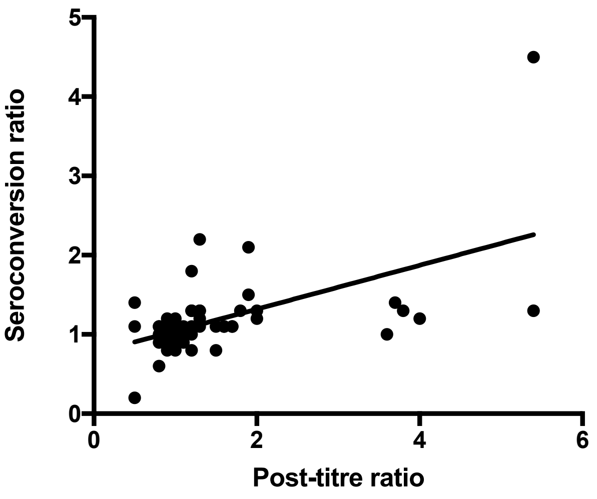

iv. **Figure S3:** Risk of bias summary for all studies (generated using Review Manager version 5.3.5)

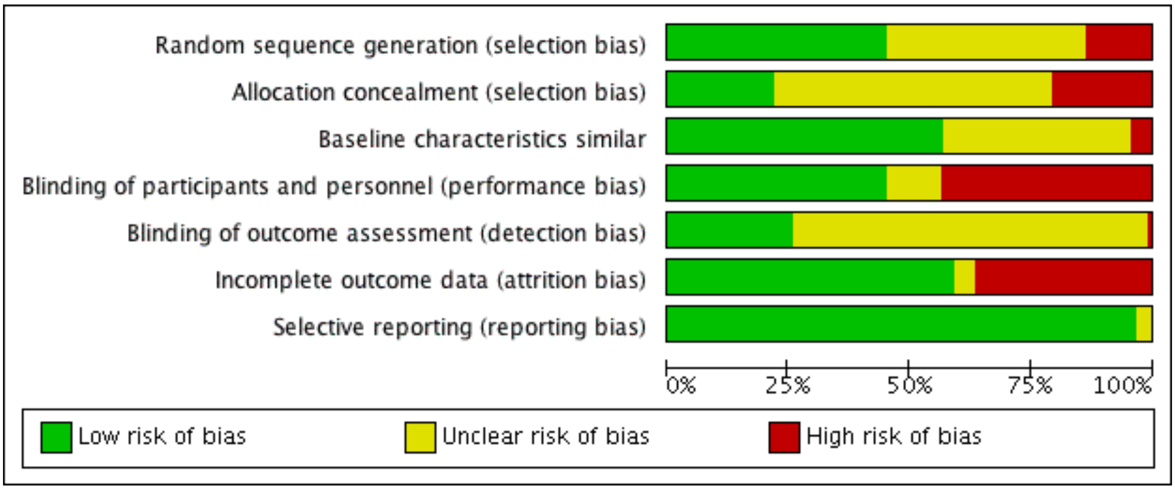

v. Forest plots and funnel plots for all interventions

Full details of the meta-analysis including all statistical outputs, code and input data can be found in an e-appendix at the following website: https://eparker12.github.io/oral_vaccine_interventions_metaanalysis_2018/

Antihelmintics

Forest plot

Study	Country	Age (m)	Vaccine	Intervention	n/N (%)	Control	n/N (%)	RR (95% CI)
**Cholera**								
Bruckner (2016)	Gabon	7.4	2x WC-rBS	Albendazole (400mg)	18/38 (47%)	Placebo	17/33 (52%)	0.92 (0.57-1.47)
Cooper (2000)	Ecuador	9	1x CVD103-HgR	Albendazole (400mg)	22/75 (29%)	Placebo	10/64 (16%)	1.88 (0.96-3.67)
**Summary**								**1.26 (0.63-2.53)**

Heterogeneity (Q 2.92, df 1), p = 0.087

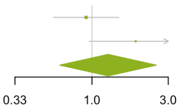

Funnel plot

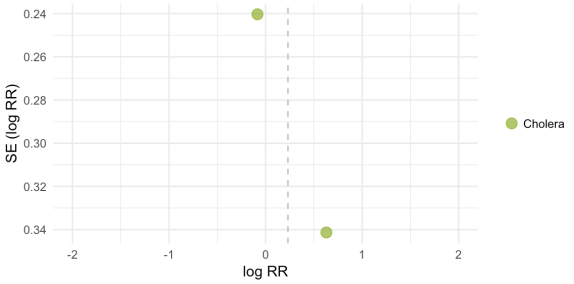

Breastfeeding withheld

Forest plot

Study	Country	Age (m)	Vaccine	Intervention	n/N (%)	Control	n/N (%)	RR (95% CI)
**Cholera**								
Ahmed (Jan 2009)	Bangladesh	7.5	2x WC-rBS	W/h BF (3h)	18/33 (55%)	U/r BF	27/47 (57%)	0.95 (0.64-1.41)
Ahmed (Jan 2009)	Bangladesh	14	2x WC-rBS	W/h BF (3h)	24/31 (77%)	U/r BF	27/49 (55%)	1.41 (1.02-1.93)
**Rotavirus**								
Ali (2015)	Pakistan	1.4	3x RV1	W/h BF (2h)	51/181 (28%)	U/r BF	65/172 (38%)	0.75 (0.55-1.01)
Ali (2015)	Pakistan	1.4	2x RV1	W/h BF (2h)	30/181 (17%)	U/r BF	50/172 (29%)	0.57 (0.38-0.85)
Groome (2014)	South Africa	1.4	2x RV1	W/h BF (2h)	62/98 (63%)	U/r BF	63/106 (59%)	1.06 (0.86-1.32)
Rongsen (2014)	India	1.6	2x RV1	W/h BF (30m)	45/172 (26%)	U/r BF	49/184 (27%)	0.98 (0.69-1.39)
**Summary**								**0.84 (0.64-1.10)**
**OPV3**								
John (1976)	India	1.5-12.8	3x tOPV	W/h BF (10h)	11/17 (65%)	U/r BF	13/18 (72%)	0.90 (0.57-1.41)
**Overall**								**0.93 (0.75-1.14)**

Heterogeneity among vaccines (Qm 1.97, df 2), p = 0.374; Residual heterogeneity (Qe 11.24, df 4), p = 0.024

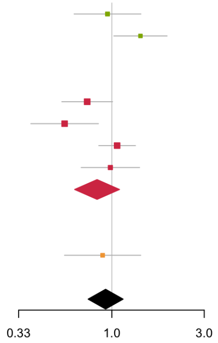

Funnel plot

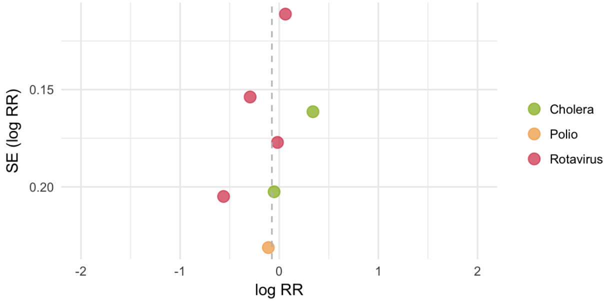

Buffer

Forest plot

Study	Country	Age (m)	Vaccine	Intervention	n/N (%)	Control	n/N (%)	RR (95% CI)
**Cholera**								
Ahmed (Jan 2009)	Bangladesh	7.5	2x WC-rBS	20ml buffer	28/49 (57%)	No buffer	7/16 (44%)	1.31 (0.71-2.39)
Ahmed (Jan 2009)	Bangladesh	14	2x WC-rBS	20ml buffer	27/49 (55%)	No buffer	6/16 (38%)	1.47 (0.74-2.90)
Sack (1997) *	USA	Adult	1x OCV Peru-15	150ml buffer	10/10 (100%)	No buffer	4/9 (44%)	2.12 (1.06-4.26)
Trach (2002)	Vietnam	Adult	2x biv-WC	150ml buffer	20/35 (57%)	No buffer	19/35 (54%)	1.05 (0.69-1.60)
**Summary**								**1.32 (0.98-1.78)**
**Rotavirus**								
Ceyhan (1993)	Turkey	1-3	1x RRV-TV	30ml buffer	60/92 (65%)	No buffer	63/102 (62%)	1.06 (0.85-1.31)
Clark (2003)	USA	2-4	3x HRRV	1ml buffer	96/108 (89%)	No buffer	107/119 (90%)	0.99 (0.90-1.08)
Ing (1991) **	USA	1.8-4	1x RRV-TV	25ml buffer	23/47 (49%)	No buffer	10/44 (23%)	2.15 (1.16-4.00)
Kerdpanich (2010)	Thailand	2.2	2x RV1	1ml buffer	133/157 (85%)	No buffer	121/154 (79%)	1.08 (0.97-1.20)
**Summary**								**1.04 (0.96-1.13)**
**OPV3**								
Chandir (2014)	Bangladesh	6.3	3x tOPV	5ml buffer	119/132 (90%)	No buffer	115/129 (89%)	1.01 (0.93-1.10)
**Overall**								**1.03 (0.98-1.09)**

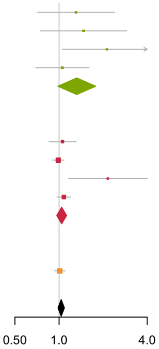

Heterogeneity among vaccines (Qm 2.80, df 2), p = 0.246; Residual heterogeneity (Qe 10.00, df 6), p = 0.125

Funnel plot

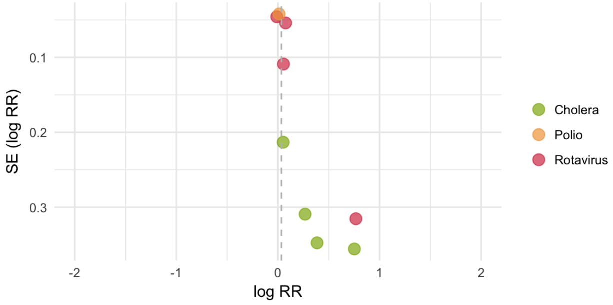

Delayed first dose

Forest plot

Study	Country	Age (m)	Vaccine	Intervention	n/N (%)	Control	n/N (%)	RR (95% CI)
**Rotavirus**								
Ali (2014)	Pakistan	1.4	2x RV1	Late (10,14w)	60/156 (38%)	Std (6,10w)	46/155 (30%)	1.30 (0.95-1.77)
Anh (2011)	Phillippines	2	2x RV1	Late (11,15w)	84/120 (70%)	Std (7,15w)	71/120 (59%)	1.18 (0.98-1.43)
Armah (2016)	Ghana	1.4	2x RV1	Late (10,14w)	52/139 (37%)	Std (6,10w)	29/142 (20%)	1.83 (1.24-2.70)
Steele (2010) *	South Africa	1.5	2x RV1 + OPV	Late (10,14w)	30/49 (61%)	Std (6,10w)	24/67 (36%)	1.71 (1.16-2.53)
Steele (2010) *	South Africa	1.5	2x RV1 + IPV	Late (10,14w)	28/51 (55%)	Std (6,10w)	28/65 (43%)	1.27 (0.88-1.85)
**Summary**								**1.37 (1.16-1.62)**

Heterogeneity (Q 5.73, df 4), p = 0.220

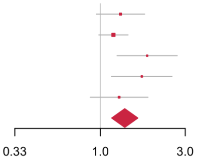

Funnel plot

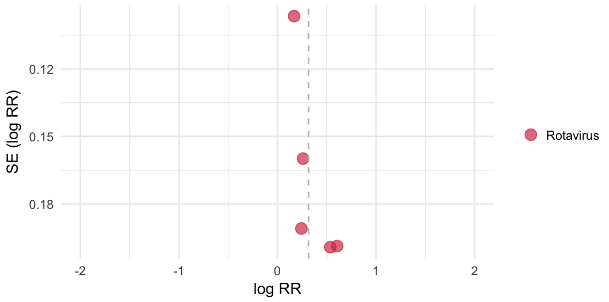

Extra dose at birth

Forest plot

Study	Country	Age (m)	Vaccine	Intervention	n/N (%)	Control	n/N (%)	RR (95% CI)
**Rotavirus**								
Bines (2015)	New Zealand	0.1	RV3-BB	3 doses	18/26 (69%)	2 doses	15/20 (75%)	0.92 (0.64-1.32)
**OPV3**								
DeXiang (1986)	China	0.1	tOPV	4 doses	107/107 (100%)	3 doses	106/107 (99%)	1.01 (0.98-1.04)
Jain (1997)	India	0	tOPV	4 doses	18/25 (72%)	3 doses	14/30 (47%)	1.54 (0.98-2.43)
Khare (1993)	India	0	tOPV	4 doses	26/30 (87%)	3 doses	32/41 (78%)	1.11 (0.90-1.38)
Osei-Kwasi (1995)	Ghana	0	tOPV	4 doses	166/200 (83%)	3 doses	155/196 (79%)	1.05 (0.95-1.15)
Weckx (1992)	Brazil	0	tOPV	4 doses	26/27 (96%)	3 doses	20/27 (74%)	1.30 (1.03-1.64)
**Summary**								**1.08 (0.99-1.18)**
**Overall**								**1.06 (0.98-1.14)**

Heterogeneity among vaccines (Qm 0.61, df 1), p = 0.436; Residual heterogeneity (Qe 8.81, df 4), p = 0.066

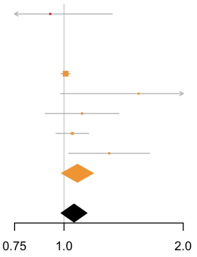

Funnel plot

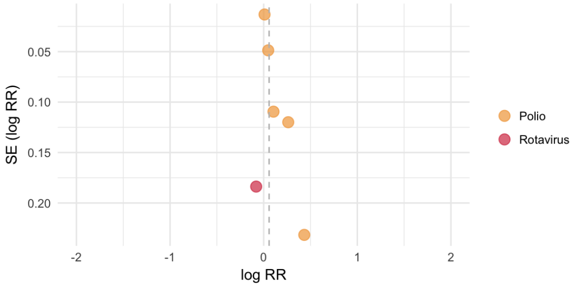

Extra dose(s)

Forest plot

Study	Country	Age (m)	Vaccine	Intervention	n/N (%)	Control	n/N (%)	RR (95% CI)
**Cholera**								
Su-Arehawaratana (1992)	Thailand	Adult	CVD103-HgR	2 doses	23/40 (58%)	1 dose	17/40 (43%)	1.35 (0.86-2.12)
**Rotavirus**								
Ali (2014)	Pakistan	1.4	RV1	6/10/14w	62/169 (37%)	10/14w *	60/156 (39%)	0.95 (0.72-1.26)
Armah (2016)	Ghana	1.4	RV1	6/10/14w	62/143 (43%)	10/14w *	52/139 (37%)	1.16 (0.87-1.54)
Kompithra (2014)	India	1.4-1.6	RV1	6/10/14/18/22w	12/44 (27%)	6/10/14w	15/44 (34%)	0.80 (0.42-1.51)
Lanata (1989)	Peru	2-18	RIT4237	3 doses	10/71 (14%)	1 dose	20/75 (27%)	0.53 (0.27-1.05)
Madhi (2010) **	Malawi	1.4	RV1	6/10/14w	24/42 (57%)	10/14w	20/42 (47%)	1.20 (0.80-1.81)
Madhi (2010) **	South Africa	1.4	RV1	6/10/14w	44/66 (67%)	10/14w	40/70 (57%)	1.17 (0.90-1.52)
Steele (JID 2010)	South Africa	1.6	RV1	6/10/14w	59/133 (44%)	10/14w	58/131 (44%)	1.00 (0.76-1.31)
**Summary**								**1.04 (0.92-1.18)**
**OPV3**								
Jhala (1981)	India	3-36	tOPV	<=6 doses	20/23 (87%)	3 doses	49/87 (56%)	1.54 (1.21-1.97)
**Overall**								**1.12 (0.96-1.30)**

Heterogeneity among vaccines (Qm 8.49, df 2), p = 0.014; Residual heterogeneity (Qe 6.58, df 6), p = 0.361

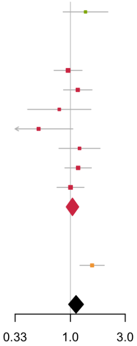

Funnel plot

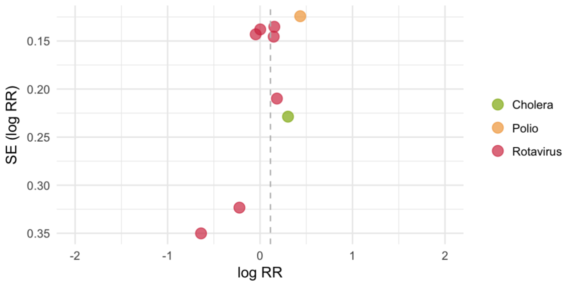

Increased vaccine inoculum

Forest plot

Study	Country	Age (m)	Vaccine	Intervention	n/N (%)	Control	n/N (%)	RR (95% CI)
**Cholera**								
Gotuzzo (1993)	Peru	Adult	1x CVD103-HgR	High (10^9)	28/39 (72%)	Std (10^8)	19/39 (49%)	1.47 (1.01-2.15)
Sow (2017)	Mali	Adult	1x CVD103-HgR	High (2x10^9)	40/48 (83%)	Std (2x10^8)	33/46 (72%)	1.16 (0.93-1.45)
Su-Arehawaratana (1992)	Thailand	Adult	1x CVD103-HgR	High (5x10^9)	17/40 (43%)	Std (5x10^8)	13/39 (33%)	1.28 (0.72-2.26)
Suharyono (1992)	Indonesia	60-108	1x CVD103-HgR	High (10^10) (c)	30/33 (91%)	Std (5x10^9)	27/31 (87%)	1.04 (0.88-1.24)
Suharyono (1992)	Indonesia	60-108	1x CVD103-HgR	High (10^10) (f)	26/32 (81%)	Std (5x10^9)	21/28 (75%)	1.08 (0.83-1.42)
**Summary**								**1.12 (1.00-1.26)**
**OPV3**								
Agarwal (1991)	India	6-12	3x tOPV	High (0.2ml)	21/35 (60%)	Std (0.1ml)	24/42 (57%)	1.05 (0.72-1.53)
Chopra (1989)	India	1-12	5x tOPV	High (0.4ml)	27/28 (96%)	Std (0.2ml)	33/34 (97%)	0.99 (0.91-1.09)
Jhala (1981)	India	3-36	3x tOPV	High (0.4ml)	14/25 (56%)	Std (0.2ml)	49/87 (56%)	0.99 (0.67-1.47)
Patriarca (1988)	Brazil	9.6	1x tOPV	High (6x10^5)	31/73 (42%)	Std (3x10^5)	13/83 (16%)	2.71 (1.54-4.78)
WHO (1995)	Brazil	0	4x tOPV	High (6x10^5)	125/195 (64%)	Std (3x10^5)	111/176 (63%)	1.02 (0.87-1.19)
WHO (1995)	Gambia	0-1.5	4x tOPV	High (6x10^5)	115/161 (71%)	Std (3x10^5)	111/159 (70%)	1.02 (0.89-1.18)
**Summary**								**1.02 (0.95-1.09)**
**Overall**								**1.05 (0.99-1.11)**

Heterogeneity among vaccines (Qm 2.08, df 1), p = 0.149; Residual heterogeneity (Qe 14.84, df 9), p = 0.096

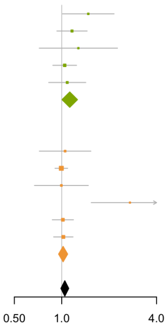

Funnel plot

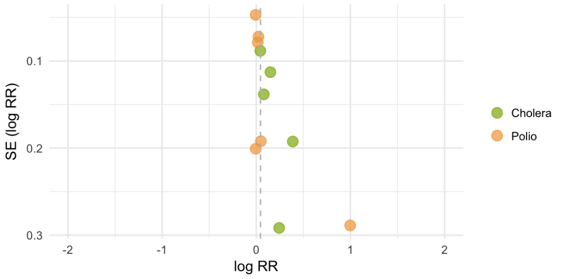

Narrow dose interval

Forest plot

Study	Country	Age (m)	Vaccine	Intervention	n/N (%)	Control	n/N (%)	RR (95% CI)
**Rotavirus**								
Anh (2011)	Vietnam	2	2x RV1	4w interval	73/130 (56%)	8w interval	73/130 (56%)	1.00 (0.81-1.24)
**OPV3**								
Cohen-Abbo (1995)	USA	6-10	3x tOPV	4w interval	25/26 (96%)	8w interval	25/25 (100%)	0.96 (0.87-1.07)
Estivariz (2015)	Bangladesh	1.4	3x bOPV	2w interval	175/186 (94%)	4w interval	176/184 (96%)	0.98 (0.94-1.03)
**Overall**								**0.98 (0.94-1.02)**

Heterogeneity among vaccines (Qm 0.03, df 1), p = 0.858; Residual heterogeneity (Qe 0.13, df 1), p = 0.720

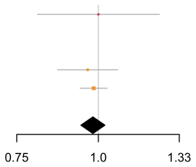

Funnel plot

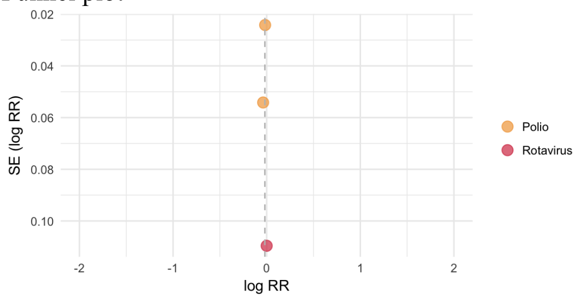

OPV valence

Forest plot (OPV-specific analysis)

Study	Country	Age (m)	Vaccine	Intervention	n/N (%)	Control	n/N (%)	RR (95% CI)
**OPV1**								
Asturias (2016)	Multiple	1.4	3x OPV	bOPV	197/198 (99%)	tOPV	86/88 (98%)	1.02 (0.98-1.05)
El-Sayed (2008)	Egypt	0	1x OPV	mOPV1	128/231 (55%)	tOPV	61/190 (32%)	1.73 (1.36-2.19)
John (1976)	India	1.5-10.3	1x OPV	mOPV1	16/18 (89%)	tOPV	11/26 (42%)	2.10 (1.30-3.39)
John (2011)	India	0	2x OPV	mOPV1	108/124 (87%)	tOPV	80/143 (56%)	1.56 (1.33-1.83)
John (2011)	India	0	1x OPV	mOPV1	27/173 (16%)	tOPV	18/176 (10%)	1.53 (0.87-2.67)
Saleem (2017)	Pakistan	0	4x OPV	bOPV	140/144 (97%)	tOPV	126/134 (94%)	1.03 (0.98-1.09)
Sutter (2010) *	India	0	2x OPV	mOPV1	151/168 (90%)	tOPV	106/168 (63%)	1.42 (1.26-1.62)
Waggie (2012)	South Africa	0	1x OPV	mOPV1	141/192 (73%)	tOPV	72/184 (39%)	1.88 (1.54-2.29)
**Summary**								**1.43 (1.19-1.74)**
**OPV3**								
Asturias (2016)	Multiple	1.4	3x OPV	bOPV	195/198 (98%)	tOPV	87/88 (99%)	1.00 (0.97-1.02)
Patriarca (1988)	Brazil	9.6	1x OPV	mOPV3	41/79 (52%)	tOPV	13/83 (16%)	3.31 (1.93-5.70)
Saleem (2017)	Pakistan	0	4x OPV	bOPV	135/144 (94%)	tOPV	114/134 (85%)	1.10 (1.01-1.20)
Sutter (2000)	Oman	9	1x OPV	mOPV3	189/205 (92%)	tOPV	155/177 (88%)	1.05 (0.98-1.13)
Sutter (2010) *	India	0	2x OPV	mOPV3	139/165 (84%)	tOPV	87/168 (52%)	1.63 (1.39-1.91)
Waggie (2012)	South Africa	0	1x OPV	mOPV3	113/195 (58%)	tOPV	39/184 (21%)	2.73 (2.02-3.70)
**Summary**								**1.54 (1.04-2.28)**
**Overall**								**1.51 (1.20-1.91)**

Heterogeneity among vaccines (Qm 0.01, df 1), p = 0.930; Residual heterogeneity (Qe 203.58, df 12), p < 0.0001

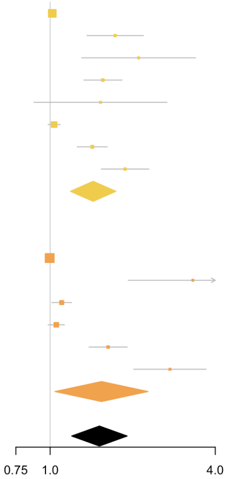

Funnel plot

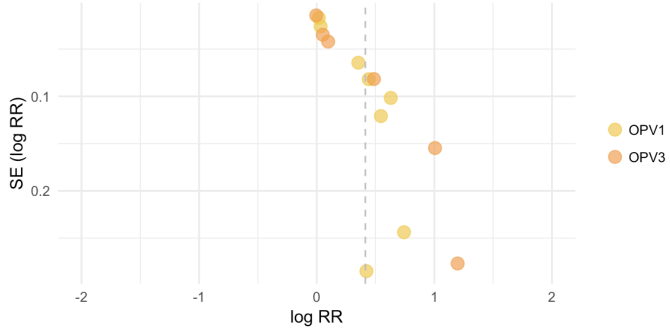

Probiotic

Forest plot

Study	Country	Age (m)	Vaccine	Intervention	n/N (%)	Control	n/N (%)	RR (95% CI)
**Cholera**								
Matsuda (2011)	Bangladesh	44.1	2x WC-rBS	Bbb01 (10^9)	40/64 (63%)	Placebo	47/62 (76%)	0.82 (0.65-1.04)
**Rotavirus**								
Isolauri (1995)	Finland	4.1	1x DxRRV	Lc ATCC	26/28 (93%)	Placebo	20/27 (74%)	1.25 (0.98-1.60)
Lazarus (2017)	India	1.2	1x RV1	LGG	42/136 (31%)	Placebo	37/135 (27%)	1.13 (0.78-1.64)
**OPV3**								
DeVrese (2005) *	Germany	Adult	1x OPV3	LGG	12/21 (57%)	Placebo	8/22 (36%)	1.57 (0.81-3.06)
**Overall**								**1.09 (0.84-1.41)**

Heterogeneity among vaccines (Qm 7.35, df 2), p = 0.025; Residual heterogeneity (Qe 0.22, df 1), p = 0.639

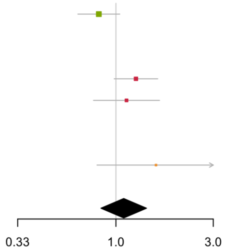

Funnel plot

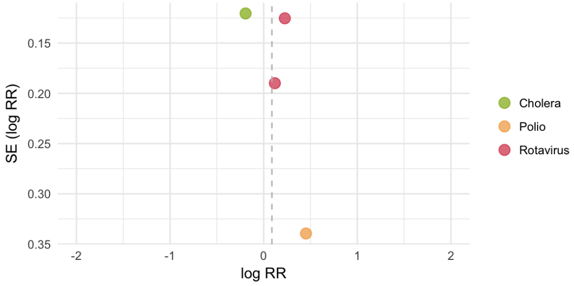

RVV separated from OPV

Forest plot (full analysis)

Study	Country	Age (m)	Vaccine	Intervention	n/N (%)	Control	n/N (%)	RR (95% CI)
**Rotavirus**								
Ciarlet (2008)	Multiple	2	3x RV5	w/o OPV	319/326 (98%)	w/ OPV	343/368 (93%)	1.05 (1.02-1.08)
Giammanco (1988)	Italy	3	1x RIT4237	w/ placebo	14/24 (58%)	w/ OPV	2/23 (9%)	6.71 (1.71-26.31)
Hanlon (1987)	Gambia	2.5	3x RIT4237	w/IPV	46/99 (46%)	w/ OPV	38/86 (44%)	1.05 (0.77-1.45)
Migasena (1995)	Thailand	2-6	3x RRV-TV	w/ placebo	25/91 (27%)	w/ OPV	15/89 (17%)	1.63 (0.92-2.88)
Steele (2010) *	South Africa	1.5	RV1 (6,10w)	w/ placebo	28/65 (43%)	w/ OPV	24/67 (36%)	1.20 (0.79-1.84)
Steele (2010) *	South Africa	1.5	RV1 (10,14w)	w/ placebo	28/51 (55%)	w/ OPV	30/49 (61%)	0.90 (0.64-1.25)
Vodopija (1986)	Yugoslavia	3	1x RIT4237	w/ placebo	19/29 (66%)	w/ OPV	10/38 (26%)	2.49 (1.37-4.51)
Zaman (2009)	Bangladesh	1.4	2x RV1	w/o OPV	44/66 (67%)	w/ OPV	39/69 (57%)	1.18 (0.90-1.54)
**Summary**								**1.21 (1.00-1.47)**
**OPV3**								
Ciarlet (2008)	Multiple	2	3x tOPV	w/o RVV	321/326 (98%)	w/ RVV	362/368 (98%)	1.00 (0.98-1.02)
Giammanco (1988)	Italy	3	1x tOPV	w/ placebo	11/21 (52%)	w/ RVV	6/23 (26%)	2.01 (0.90-4.47)
Hanlon (1987)	Gambia	2.5	3x tOPV	w/ placebo	57/95 (60%)	w/ RVV	45/87 (52%)	1.16 (0.89-1.51)
Steele (2010)	South Africa	1.5	2x tOPV	w/ placebo	61/62 (99%)	w/ RVV	49/50 (98%)	1.00 (0.95-1.06)
Vodopija (1986)	Yugoslavia	3	1x tOPV	w/ placebo	12/33 (36%)	w/ RVV	11/29 (38%)	0.96 (0.50-1.83)
Zaman (2009)	Bangladesh	1.4	3x tOPV	w/o RVV	22/36 (61%)	w/ RVV	48/69 (70%)	0.88 (0.65-1.19)
**Summary**								**1.00 (0.98-1.02)**
**Overall**								**1.01 (1.00-1.03)**

Heterogeneity among vaccines (Qm 8.20, df 1), p = 0.004; Residual heterogeneity (Qe 24.20, df 12), p = 0.019

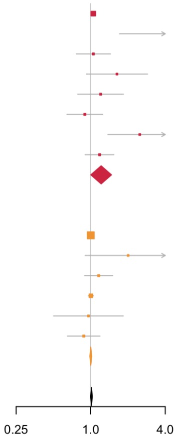

Funnel plot

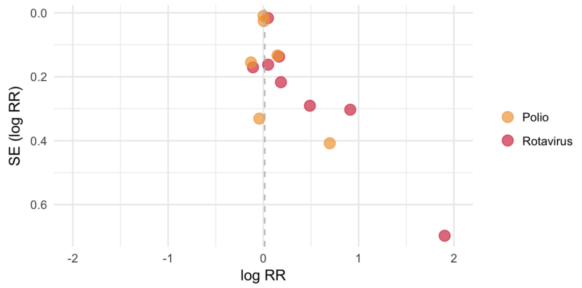

Forest plot (OPV-specific analysis)

Study	Country	Age (m)	Vaccine	Intervention	n/N (%)	Control	n/N (%)	RR (95% CI)
**OPV1**								
Ciarlet (2008)	Multiple	2	3x tOPV	w/o RVV	324/326 (99%)	w/ RVV	364/368 (99%)	1.00 (0.99-1.02)
Giammanco (1988)	Italy	3	1x tOPV	w/ placebo	18/21 (86%)	w/ RVV	16/23 (70%)	1.23 (0.89-1.70)
Hanlon (1987)	Gambia	2.5	3x tOPV	w/ placebo	79/95 (83%)	w/ RVV	68/87 (78%)	1.06 (0.92-1.23)
Migasena (1995)	Thailand	2-6	3x tOPV	w/ placebo	86/94 (91%)	w/ RVV	74/88 (84%)	1.09 (0.97-1.21)
Steele (2010) **	South Africa	1.5	2x tOPV	w/ placebo	68/68 (100%)	w/ RVV	52/52 (100%)	1.00 (0.97-1.04)
Vodopija (1986)	Yugoslavia	3	1x tOPV	w/ placebo	21/35 (60%)	w/ RVV	21/33 (64%)	0.94 (0.65-1.37)
Zaman (2009)	Bangladesh	1.4	3x tOPV	w/o RVV	33/35 (94%)	w/ RVV	57/66 (86%)	1.09 (0.96-1.24)
**Summary**								**1.01 (0.99-1.02)**
**OPV2**								
Ciarlet (2008)	Multiple	2	3x tOPV	w/o RVV	325/326 (100%)	w/ RVV	367/368 (100%)	1.00 (0.99-1.01)
Hanlon (1987)	Gambia	2.5	3x tOPV	w/ placebo	90/95 (95%)	w/ RVV	82/87 (94%)	1.01 (0.94-1.08)
Steele (2010) **	South Africa	1.5	2x tOPV	w/ placebo	68/68 (100%)	w/ RVV	52/52 (100%)	1.00 (0.97-1.04)
Zaman (2009)	Bangladesh	1.4	3x tOPV	w/o RVV	33/34 (97%)	w/ RVV	67/68 (99%)	0.99 (0.92-1.05)
**Summary**								**1.00 (0.99-1.01)**
**OPV3**								
Ciarlet (2008)	Multiple	2	3x tOPV	w/o RVV	321/326 (98%)	w/ RVV	362/368 (98%)	1.00 (0.98-1.02)
Giammanco (1988)	Italy	3	1x tOPV	w/ placebo	11/21 (52%)	w/ RVV	6/23 (26%)	2.01 (0.90-4.47)
Hanlon (1987)	Gambia	2.5	3x tOPV	w/ placebo	57/95 (60%)	w/ RVV	45/87 (52%)	1.16 (0.89-1.51)
Steele (2010) **	South Africa	1.5	2x tOPV	w/ placebo	61/62 (99%)	w/ RVV	49/50 (98%)	1.00 (0.95-1.06)
Vodopija (1986)	Yugoslavia	3	1x tOPV	w/ placebo	12/33 (36%)	w/ RVV	11/29 (38%)	0.96 (0.50-1.83)
Zaman (2009)	Bangladesh	1.4	3x tOPV	w/o RVV	22/36 (61%)	w/ RVV	48/69 (70%)	0.88 (0.65-1.19)
**Summary**								**1.00 (0.98-1.02)**
**Overall**								**1.00 (1.00-1.01)**

Heterogeneity among vaccines (Qm 0.95, df 2), p = 0.623; Residual heterogeneity (Qe 10.97, df 14), p = 0.689

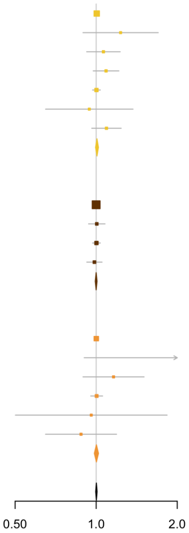

Vitamin A

Forest plot (full analysis)

Study	Country	Age (m)	Vaccine	Intervention	n/N (%)	Control	n/N (%)	RR (95% CI)
**Cholera**								
Albert (2003)	Bangladesh	40	2x Cholerix	Vit A (200k IU)	34/61 (56%)	Placebo	29/63 (46%)	1.21 (0.85-1.72)
**OPV3**								
Bahl (2002)	India	0.8	3x tOPV	Vit A (25k IU)	164/194 (85%)	Placebo	165/205 (80%)	1.05 (0.96-1.15)
Newton (2005)	Ghana	0.9	3x tOPV	Vit A (25k IU)	171/192 (89%)	Placebo	171/194 (88%)	1.01 (0.94-1.09)
Rahman (1998)	Bangladesh	1.5-4.3	3x tOPV	Vit A (50k IU)	28/34 (82%)	Placebo	20/23 (87%)	0.95 (0.76-1.18)
Semba (1999)	Indonesia	1.7	3x tOPV	Vit A (50k IU)	113/113 (100%)	Placebo	111/112 (99%)	1.01 (0.98-1.03)
**Summary**								**1.01 (0.99-1.03)**
**Overall**								**1.01 (0.99-1.03)**

Heterogeneity among vaccines (Qm 1.02, df 1), p = 0.311; Residual heterogeneity (Qe 1.04, df 3), p = 0.791

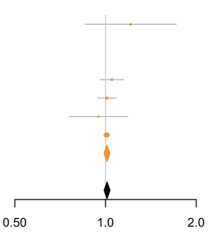

Funnel plot

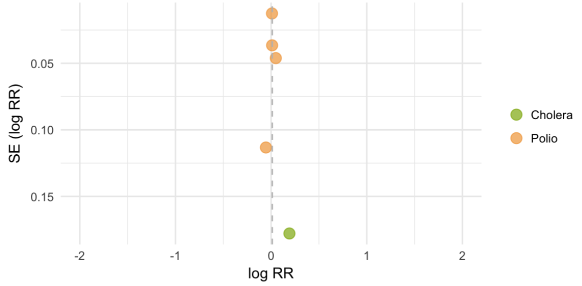

Forest plot (OPV-specific analysis)

Study	Country	Age (m)	Vaccine	Intervention	n/N (%)	Control	n/N (%)	RR (95% CI)
**OPV1**								
Bahl (2002)	India	0.8	3x tOPV	Vit A (25k IU)	159/194 (82%)	Placebo	146/205 (71%)	1.15 (1.03-1.28)
Newton (2005)	Ghana	0.9	3x tOPV	Vit A (25k IU)	174/192 (91%)	Placebo	181/194 (93%)	0.97 (0.92-1.03)
Rahman (1998)	Bangladesh	1.5-4.3	3x tOPV	Vit A (50k IU)	27/34 (79%)	Placebo	19/23 (83%)	0.96 (0.75-1.24)
Semba (1999)	Indonesia	1.7	3x tOPV	Vit A (50k IU)	100/102 (98%)	Placebo	91/92 (99%)	0.99 (0.96-1.03)
**Summary**								**1.02 (0.94-1.10)**
**OPV2**								
Bahl (2002)	India	0.8	3x tOPV	Vit A (25k IU)	179/194 (92%)	Placebo	191/205 (93%)	0.99 (0.94-1.05)
Newton (2005)	Ghana	0.9	3x tOPV	Vit A (25k IU)	181/192 (94%)	Placebo	183/194 (94%)	1.00 (0.95-1.05)
Rahman (1998)	Bangladesh	1.5-4.3	3x tOPV	Vit A (50k IU)	28/34 (82%)	Placebo	21/23 (91%)	0.90 (0.74-1.10)
Semba (1999)	Indonesia	1.7	3x tOPV	Vit A (50k IU)	116/116 (100%)	Placebo	113/113 (100%)	1.00 (0.98-1.02)
**Summary**								**1.00 (0.98-1.01)**
**OPV3**								
Bahl (2002)	India	0.8	3x tOPV	Vit A (25k IU)	164/194 (85%)	Placebo	165/205 (80%)	1.05 (0.96-1.15)
Newton (2005)	Ghana	0.9	3x tOPV	Vit A (25k IU)	171/192 (89%)	Placebo	171/194 (88%)	1.01 (0.94-1.09)
Rahman (1998)	Bangladesh	1.5-4.3	3x tOPV	Vit A (50k IU)	28/34 (82%)	Placebo	20/23 (87%)	0.95 (0.76-1.18)
Semba (1999)	Indonesia	1.7	3x tOPV	Vit A (50k IU)	113/113 (100%)	Placebo	111/112 (99%)	1.01 (0.98-1.03)
**Summary**								**1.01 (0.99-1.03)**
**Overall**								**1.00 (0.99-1.01)**

Heterogeneity among vaccines (Qm 0.94, df 2), p = 0.626; Residual heterogeneity (Qe 9.71, df 9), p = 0.374

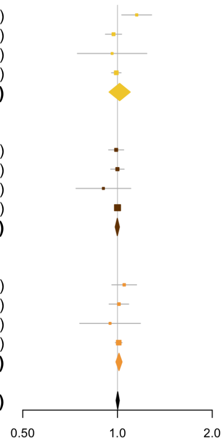

Zinc

Forest plot

Study	Country	Age (m)	Vaccine	Intervention	n/N (%)	Control	n/N (%)	RR (95% CI)
**Cholera**								
Ahmed (Jan 2009)	Bangladesh	7.5	2x WC-rBS	Zinc (20mg)	11/36 (31%)	No Zinc	27/47 (57%)	0.53 (0.31-0.92)
Ahmed (Jan 2009)	Bangladesh	14	2x WC-rBS	Zinc (20mg)	27/34 (79%)	No Zinc	27/49 (55%)	1.44 (1.06-1.96)
Ahmed (Oct 2009)	Bangladesh	14	2x WC-rBS	Zinc (20mg)	15/18 (83%)	No Zinc	11/20 (55%)	1.52 (0.97-2.37)
Albert (2003)	Bangladesh	40	2x Cholerix	Zinc (20mg)	39/63 (62%)	Placebo	29/63 (46%)	1.34 (0.97-1.87)
**Summary**								**1.16 (0.75-1.79)**
**Rotavirus**								
Lazarus (2017)	India	1.2	2x RV1	Zinc (5mg)	40/143 (28%)	Placebo	37/135 (27%)	1.02 (0.70-1.49)
**OPV3**								
Habib (2015)	Pakistan	0.3	4x tOPV	Zinc (10mg)	94/156 (60%)	Placebo	99/163 (61%)	0.99 (0.83-1.18)
**Overall**								**1.11 (0.87-1.42)**

Heterogeneity among vaccines (Qm 0.15, df 2), p = 0.927; Residual heterogeneity (Qe 10.97, df 3), p = 0.012

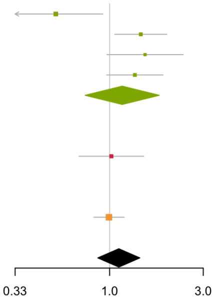

Funnel plot

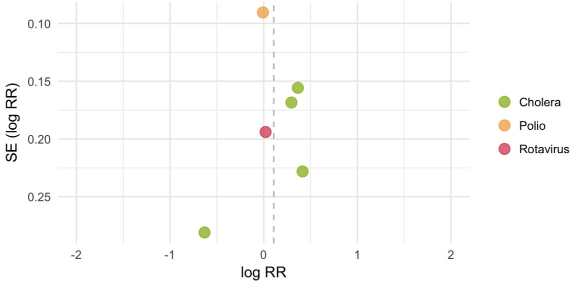

a. Footnotes for Forest plots (above)

	Intervention	Study	Notes
1	Buffer	Sack (1997) *	This study also examined another buffer, CeraVacx (sodium bicarbonate, trisodium citrate, rice syrup) with identical results. We excluded these data from the forest plot to avoid replication of the control group.
2	Buffer	Ing (1991) **	This study also examined the same buffer at a smaller volume (2ml). We excluded these data from the forest plot to avoid replication of the control group.
3	Delayed first dose	Steele (2010) *	Intervention and control arm recruited separately. Exact sample sizes were not reported for immunogenicity data and were therefore estimated by assuming that loss-to-follow-up rates reported in Figure 1 of the trial report were evenly distributed across arms.
3	Extra dose(s)	10/14 *	Study included both a 6/10w and 10/14w dose schedule. The 10/14w schedule was selected as the control group to ensure consistency with other studies and to delineate the effect of extra doses from delayed doses (considered in a separate comparison).
4	Extra dose(s)	Madhi (2010) **	Immunogenicity data extracted from Madhi et al. (Vaccine 2012) and Cunliffe et al. (Vaccine 2012). Exact sample sizes were not reported for Malawi data; we therefore assumed that the 85 RVV recipients were distributed 1:1 across the 2-dose and 3-dose schedules (n = 42 per arm) and used the reported seroconversion rates (47.2% and 57.1%) to estimate the number of infants who seroconverted.
5	OPV valency	Sutter (2010) *	This study also included an arm comparing bOPV with tOPV. We excluded these data from the forest plot to avoid replication of the control group.
6	Probiotic	De Vrese (2005) *	This study also examined another probiotic, Lactobacillus casei CRL431 10^10 cfu, with similar results. We excluded these data from the forest plot to avoid replication of the control group.
8	RVV separated from OPV	Steele (2010) *	Exact sample sizes were not reported for immunogenicity data and were therefore estimated by assuming that loss-to-follow-up rates reported in Figure 1 of the trial report were evenly distributed across arms.

b. Acronyms for Forest plots (above)

Acronym	Notes
1h	One hour
4w	Four weeks
50k	50,000
Bbb01	Bifidobacterium breve 01
BF	Breastfeeding
(c)	Centrifuged
(f)	Filtered
IPV	Inactivated poliovirus vaccine
IU	International Units
Lc ATCC	Lactobacillus casei ATCC
LGG	Lactobacillus GG
OCV	Oral cholera vaccine
OPV	Oral poliovirus vaccine
bOPV	Bivalent OPV
mOPV	Monovalent OPV
tOPV	Trivalent OPV
RVV	Rotavirus vaccine
RV1	Rotarix monovalent vaccine
RV5	RotaTeq pentavalent vaccine
Ty21a	Live attenuated strain of salmonella typhi
U/r	Unrestricted
Vit A	Vitamin A
w/	With
w/h	Withheld
w/o	Without

vi. **Table S4:** Summary of secondary analyses for each intervention category included in the meta-analysis, describing 1) moderator effect according to vaccine type, 2) heterogeneity left over after accounting for moderator effect, and 3) funnel pot asymmetry.

Intervention	Moderator effect[4] (P value)	Residual heterogeneity (P value)	Egger's test[5] (P value)	No. of studies in meta-regression (K)[6]
Antihelminthic		0.087		2 (2 OCV)
Breastfeeding withheld	0.374	0.024	0.360	7 (2 OCV, 4 RV, 1 PV3)
Buffer	0.246	0.125	0.004	9 (4 OCV, 4 RV, 1 PV3)
Delayed first dose		0.221	0.053	5 (5 RV)
Extra dose at birth	0.436	0.066	0.027	6 (1 RV, 5 PV3)
Extra dose(s)	0.014	0.361	RV 0.089	9 (1 OCV, 7 RV, 1 PV3)
Increased vaccine inoculum	0.149	0.096	0.003	11 (5 OCV, 6 PV3)
Narrow dose interval	0.858	0.720	0.953	3 (1 RV, 2 PV3)
OPV valence		<0.0001	<0.0001	6 (6 PV3)
Probiotic	0.025	0.639		4 (1 OCV, 2 RV, 1 PV3)
RVV separated from OPV	0.004	0.019	RV 0.005<br>PV3 0.359	14 (8 RV, 6 PV3)
Vitamin A	0.311	0.791	0.518	5 (1 OCV, 4 PV3)
Zinc	0.927	0.012	0.439	6 (4 OCV, 1 RV, 1 PV3)

Significant effect (P value <0.05)

[4] Moderator effect, testing for heterogeneity between vaccines, is reported only if overall K ≥3 and ≥2 oral vaccine types included.

[5] Egger's regression test, measuring funnel plot asymmetry, is reported if K ≥3. If the moderator effect was significant (P value <0.05) or there was replication of infants across multiple vaccine types, we performed separate Egger's tests for each vaccine.

[6] K also refers to strata within studies

vii. **Table S5:** Summary of meta-regression results where heterogeneity not accounted for by vaccine type (i.e. residual heterogeneity P<0.05) testing age, income setting and background immunogenicity as secondary moderators.

Intervention	Age group[7] (P value)	Income setting[8] (P value)	Background immunogenicity[9] (P value)	Notes
Breastfeeding withheld	0.916	0.571	0.185	No moderators significant
OPV valence		0.626	<0.0001	Strong negative correlation with background immunogenicity
RVV separated from OPV[10]		0.542	0.016	The benefit of separate vaccine delivery for RVV seroconversion is driven primarily by studies that administered a single dose and is negatively correlated with background immunogenicity.
Zinc	0.002		0.869	Zinc has more beneficial effect on vaccine outcome in children than infants.

Significant effect (P value <0.05)

[7] Age group sub-divided into four categories: infant <1year, child 1-5 years, child 5-17 years and adult >=18years.

[8] Income setting sub-divided into low or lower-middle versus upper-middle or high.

[9] Background immunogenicity defined as the seroconversion rate in the control arm and modeled as a continuous variable after arcsine square root transformation to approximate a normal distribution.

[10] In vaccine-specific meta-analyses, residual heterogeneity was significant for RVV response but not OPV. Secondary moderators were therefore tested for RVV only.

viii. Sensitivity analysis

Due to variation in timing of post-vaccine titre measurements, we conducted a post-hoc sensitivity analysis, excluding 19 studies from the meta-analysis that measured seroconversion outside our pre-specified windows (Table S1, Appendix).

Intervention	No of studies overall	No of studies in sensitivity	Overall Effect RR [95% CI]	Sensitivity Effect RR [95% CI]
Antihelminthic	2	1	1·26 [0.63-2·53]	
Breastfeeding withheld	7	7	0·93 [0·75-1·14]	0·93 [0·75-1·14]
Buffer	9	7	1·03 [0·98-1·09]	1.02 [0.96-1.08]
Delayed first dose	5	5	1.37 [1.16-1.62]	1.37 [1.16-1.62]
Extra dose at birth	6	4	1·06 [0·98-1·14]	1.01 [0.99-1.04]
Extra dose(s)	9	8	1·12 [0·96-1·30]	1.06 [0.94-1.20]
Increased vaccine inoculum	11	7	1.05 [0.99-1.11]	1.03 [0.97-1.09]
Narrow dose interval	3	2	0·98 [0·94-1·02]	0.97 [0.88-1.07]
OPV valence	6	4	1·51 [1·20-1·91]	1.48 [1.18-1.85]
Probiotic	4	3	1·09 [0·84-1·41]	1.04 [0.80-1.37]
RVV separated from OPV[11]	14	12	1·21 [1·00-1·47]	1.33 [1.00-1.77]
Vitamin A	5	2	1·01 [0·99-1·03]	1.03 [0.82-1.30]
Zinc	6	6	1·11 [0·87-1·42]	1·11 [0·87-1·42]
**Total**	87	68		

The effect of delayed first dose of RVV remained identical to the main analysis. For OPV valence, findings were similar to the main analysis but with widened confidence intervals. Similarly, the effect of separating RVV from OPV on RVV seroconversion and the increased inoculum effect for OCV remained similar to the main meta-analysis (RR 1.09 [95% CI 0.97-1.23]. For all other interventions, there remained no evidence of impact.

[11] Effect size refers to percent RVV seroconversion outcome.

ix. **Table 1:** Overview of 87 intervention studies included in the systematic review (with references assigned)

	**Oral Vaccine**			
	**Poliovirus**	**Rotavirus**	**Cholera**	**Typhoid**
**Total studies (N)***	**46**	**24**	**15**	**9**
**Intervention**				
Antihelminthic	0	0	2[49,50]	1[51]
Antibiotic**	1[52]	0	0	0
Breastfeeding withheld	2[53,54]	3[55-57]	1[58]	0
Buffer	1[59]	4[60-63]	3[58,64,65]	3[47,66,67]
Delayed first dose	0	4[26,27,40,42]	0	0
Early first dose**	1[68]	0	0	0
Extra dose(s)	1[43]	6[26,27,69-72]	1[44]	2[73,74]
Extra dose at birth	5[75-79]	1[80]	0	0
Miscellaneous**	3[81-83]	0	1[84]	0
Narrow dose interval	3[85-87]	1[40]	0	1[47]
OPV valence	10[46,88-95]	NA	NA	NA
Other micronutrients**	2[96,97]	0	0	1[98]
Probiotic	1[48]	2[41,99]	2[100,101]	1[102]
RVV separated from OPV	7[103]	7[42,103-108]	NA	NA
Vaccine inoculum	7[25,43,45,46,109-111]	0	4[44,112-114]	0
Vitamin A	4[115-118]	0	1[39]	1[119]
Zinc	1[120]	1[41]	4[39,58,121,122]	0
**Age group**				
<1mo	19	1	0	0
1-12mo	24	23	3	0
1y-15y	2	0	4	6***
=>16y	1	0	8	3
Mean (SD) age; mo	4.2 (7.9)	1.9 (1.3)	141.6 (163.3)	187.9 (133.8)
**Sex**				
Males (%)	51.3	45.7	50.5	55.9
**Location**				
Africa	8	6	2	1
Asia	25	10	7	2
Europe	5	3	2	2
Americas	8	4	4	4
Oceania	0	1	0	0
**Study size**				
<50 participants with SC data	7	1	2	2
50-500	37	21	13	2
>500	2	2	0	5
**Total SC data (N)**	**8838**	**8954**	**1395**	**353030**

RVV = rotavirus vaccine, OPV = oral poliovirus vaccine, SC = seroconversion, mo = months, y = years

* Of 86 unique studies, some studies examined two or more interventions and some reported on multiple oral vaccine targets (Table S3).

** There were insufficient studies (<2) of antibiotics, early first dose, other micronutrients and miscellaneous interventions (maternal vitamin A, horse anti-serum, soya formula and E. coli K12) for inclusion in the meta-analysis.

*** Most of these typhoid studies recruited children aged between 5 and 22 years

Supplementary references

1. Parker EP, Ramani S, Lopman BA, et al. Causes of impaired oral vaccine efficacy in developing countries. *Future Microbiol* 2017.
2. Stevens GA, Bennett JE, Hennocq Q, et al. Trends and mortality effects of vitamin A deficiency in children in 138 low-income and middle-income countries between 1991 and 2013: a pooled analysis of population-based surveys. *Lancet Glob Health* 2015; **3**(9): e528-36.
3. WHO. Vitamin A supplementation for infants and children 6-59 months of age: Guideline, 2011.
4. Kaufman DR, De Calisto J, Simmons NL, et al. Vitamin A deficiency impairs vaccine-elicited gastrointestinal immunity. *J Immunol* 2011; **187**(4): 1877-83.
5. Surman SL, Jones BG, Sealy RE, Rudraraju R, Hurwitz JL. Oral retinyl palmitate or retinoic acid corrects mucosal IgA responses toward an intranasal influenza virus vaccine in vitamin A deficient mice. *Vaccine* 2014; **32**(22): 2521-4.
6. Athanassiades TJ. Adjuvant effect of vitamin A palmitate and analogs on cell-mediated immunity. *J Natl Cancer Inst* 1981; **67**(5): 1153-6.
7. Lisulo MM, Kapulu MC, Banda R, et al. Adjuvant potential of low dose all-trans retinoic acid during oral typhoid vaccination in Zambian men. *Clin Exp Immunol* 2014; **175**(3): 468-75.
8. Shankar AH, Prasad AS. Zinc and immune function: the biological basis of altered resistance to infection. *Am J Clin Nutr* 1998; **68**(2 Suppl): 447S-63S.
9. Bhutta ZA, Das JK, Walker N, et al. Interventions to address deaths from childhood pneumonia and diarrhoea equitably: what works and at what cost? *Lancet* 2013; **381**(9875): 1417-29.
10. Kotloff KL, Nataro JP, Blackwelder WC, et al. Burden and aetiology of diarrhoeal disease in infants and young children in developing countries (the Global Enteric Multicenter Study, GEMS): a prospective, case-control study. *Lancet* 2013; **382**(9888): 209-22.
11. Parker EP, Kampmann B, Kang G, Grassly NC. Influence of enteric infections on response to oral poliovirus vaccine: a systematic review and meta-analysis. *J Infect Dis* 2014; **210**(6): 853-64.
12. Wang J, Li F, Wei H, Lian ZX, Sun R, Tian Z. Respiratory influenza virus infection induces intestinal immune injury via microbiota-mediated Th17 cell-dependent inflammation. *J Exp Med* 2014; **211**(12): 2397-410.
13. Prendergast A, Kelly P. Enteropathies in the developing world: neglected effects on global health. *Am J Trop Med Hyg* 2012; **86**(5): 756-63.
14. Naylor C, Lu M, Haque R, et al. Environmental Enteropathy, Oral Vaccine Failure and Growth Faltering in Infants in Bangladesh. *EBioMedicine* 2015; **2**(11): 1759-66.
15. Becker-Dreps S, Vilchez S, Bucardo F, et al. The Association Between Fecal Biomarkers of Environmental Enteropathy and Rotavirus Vaccine Response in Nicaraguan Infants. *Pediatr Infect Dis J* 2017; **36**(4): 412-6.
16. Hotez PJ, Fenwick A, Savioli L, Molyneux DH. Rescuing the bottom billion through control of neglected tropical diseases. *Lancet* 2009; **373**(9674): 1570-5.
17. Hall A, Hewitt G, Tuffrey V, de Silva N. A review and meta-analysis of the impact of intestinal worms on child growth and nutrition. *Matern Child Nutr* 2008; **4 Suppl 1**: 118-236.
18. Cooper PJ, Chico M, Sandoval C, et al. Human infection with Ascaris lumbricoides is associated with suppression of the interleukin-2 response to recombinant cholera toxin B subunit following vaccination with the live oral cholera vaccine CVD 103-HgR. *Infection & Immunity* 2001; **69**(3): 1574-80.
19. WHO. Deworming to combat the health and nutritional impact of soil-transmitted helminths. *World Health Organisation* 2012.
20. Hooper LV, Littman DR, Macpherson AJ. Interactions between the microbiota and the immune system. *Science* 2012; **336**(6086): 1268-73.
21. Harris VC, Armah G, Fuentes S, et al. Significant Correlation Between the Infant Gut Microbiome and Rotavirus Vaccine Response in Rural Ghana. *J Infect Dis* 2017; **215**(1): 34-41.
22. Parker EPK, Praharaj I, Zekavati A, et al. Influence of the intestinal microbiota on the immunogenicity of oral rotavirus vaccine given to infants in south India. *Vaccine* 2018; **36**(2): 264-72.
23. Kvistgaard AS, Pallesen LT, Arias CF, et al. Inhibitory effects of human and bovine milk constituents on rotavirus infections. *J Dairy Sci* 2004; **87**(12): 4088-96.
24. Novak D, Svennerholm AM. A comparison of seasonal variations in rotavirus antibodies in the breast milk of Swedish and Bangladeshi mothers. *Acta Paediatr* 2015; **104**(3): 247-51.
25. Estivariz CF, Jafari H, Sutter RW, et al. Immunogenicity of supplemental doses of poliovirus vaccine for children aged 6-9 months in Moradabad, India: a community-based, randomised controlled trial. *Lancet Infect Dis* 2012; **12**(2): 128-35.

26. Ali SA, Kazi AM, Cortese MM, et al. Impact of different dosing schedules on the immunogenicity of the human rotavirus vaccine in infants in Pakistan: a randomized trial. *J Infect Dis* 2014; **210**(11): 1772-9.
27. Armah G, Lewis KD, Cortese MM, et al. A Randomized, Controlled Trial of the Impact of Alternative Dosing Schedules on the Immune Response to Human Rotavirus Vaccine in Rural Ghanaian Infants. *J Infect Dis* 2016; **213**(11): 1678-85.
28. Colgate ER, Haque R, Dickson DM, et al. Delayed Dosing of Oral Rotavirus Vaccine Demonstrates Decreased Risk of Rotavirus Gastroenteritis Associated With Serum Zinc: A Randomized Controlled Trial. *Clin Infect Dis* 2016; **63**(5): 634-41.
29. Gruber JF, Hille DA, Liu GF, et al. Heterogeneity of Rotavirus Vaccine Efficacy Among Infants in Developing Countries. *Pediatric Infectious Disease Journal* 2017; **36**(1): 72-8.
30. Dang DA, Nguyen VT, Vu DT, et al. A dose-escalation safety and immunogenicity study of a new live attenuated human rotavirus vaccine (Rotavin-M1) in Vietnamese children. *Vaccine* 2012; **30 Suppl 1**: A114-21.
31. Black RE, Victora CG, Walker SP, et al. Maternal and child undernutrition and overweight in low-income and middle-income countries. *Lancet* 2013; **382**(9890): 427-51.
32. Dominguez-Salas P, Moore SE, Baker MS, et al. Maternal nutrition at conception modulates DNA methylation of human metastable epialleles. *Nat Commun* 2014; **5**: 3746.
33. Malhotra I, Mungai P, Wamachi A, et al. Helminth- and Bacillus Calmette-Guerin-induced immunity in children sensitized in utero to filariasis and schistosomiasis. *J Immunol* 1999; **162**(11): 6843-8.
34. Malhotra I, Ouma JH, Wamachi A, et al. Influence of maternal filariasis on childhood infection and immunity to Wuchereria bancrofti in Kenya. *Infect Immun* 2003; **71**(9): 5231-7.
35. Webb EL, Mawa PA, Ndibazza J, et al. Effect of single-dose anthelmintic treatment during pregnancy on an infant's response to immunisation and on susceptibility to infectious diseases in infancy: a randomised, double-blind, placebo-controlled trial. *Lancet* 2011; **377**(9759): 52-62.
36. Clark CE, Fay MP, Chico ME, et al. Maternal Helminth Infection Is Associated With Higher Infant Immunoglobulin A Titers to Antigen in Orally Administered Vaccines. *J Infect Dis* 2016; **213**(12): 1996-2004.
37. Ryan R HS. How to GRADE the quality of evidence. 2016.
38. Ahmed T, Svennerholm AM, Tarique AA, Sultana GNN, Qadri F. Enhanced immunogenicity of an oral inactivated cholera vaccine in infants in Bangladesh obtained by zinc supplementation and by temporary withholding breast-feeding. *Vaccine* 2009; **27**(9): 1433-9.
39. Albert MJ, Qadri F, Wahed MA, et al. Supplementation with zinc, but not vitamin A, improves seroconversion to vibriocidal antibody in children given an oral cholera vaccine. *Journal of Infectious Diseases* 2003; **187**(6): 909-13.
40. Anh DD, Carlos CC, Thiem DV, et al. Immunogenicity, reactogenicity and safety of the human rotavirus vaccine RIX4414 (RotarixTM) oral suspension (liquid formulation) when co-administered with expanded program on immunization (EPI) vaccines in Vietnam and the Philippines in 2006-2007. *Vaccine* 2011; **29**(11): 2029-36.
41. Lazarus RP, John J, Shanmugasundaram E, et al. The effect of probiotics and zinc supplementation on the immune response to oral rotavirus vaccine: A randomized, factorial design, placebo-controlled study among Indian infants. *Vaccine* 2018; **36**(2): 273-9.
42. Steele AD, De Vos B, Tumbo J, et al. Co-administration study in South African infants of a live-attenuated oral human rotavirus vaccine (RIX4414) and poliovirus vaccines. *Vaccine* 2010; **28**(39): 6542-8.
43. Jhala CI, Goel RK. Antibody response to three doses of conventional and double-dose OPV and to six doses of conventional OPV. *Indian Pediatr* 1981; **18**(6): 379-83.
44. Su-Arehawaratana P, Singharaj P, Taylor DN, et al. Safety and immunogenicity of different immunization regimens of CVD 103-HgR live oral cholera vaccine in soldiers and civilians in Thailand. *J Infect Dis* 1992; **165**(6): 1042-8.
45. John TJ, Jain H, Ravishankar K, et al. Monovalent type 1 oral poliovirus vaccine among infants in India: report of two randomized double-blind controlled clinical trials. *Vaccine* 2011; **29**(34): 5793-801.
46. Patriarca PA, Laender F, Palmeira G, et al. Randomised trial of alternative formulations of oral poliovaccine in Brazil. *Lancet* 1988; **1**(8583): 429-33.
47. Levine MM, Ferreccio C, Black RE, Germanier R. Large-scale field trial of Ty21a live oral typhoid vaccine in enteric-coated capsule formulation. *Lancet* 1987; **1**(8541): 1049-52.
48. de Vrese M, Rautenberg P, Laue C, Koopmans M, Herremans T, Schrezenmeir J. Probiotic bacteria stimulate virus-specific neutralizing antibodies following a booster polio vaccination. *European Journal of Nutrition* 2005; **44**(7): 406-13.

49. Bruckner S, Agnandji ST, Elias J, et al. A single-dose antihelminthic treatment does not influence immunogenicity of a meningococcal and a cholera vaccine in Gabonese school children. *Vaccine* 2016; **34**(44): 5384-90.
50. Cooper PJ, Chico ME, Losonsky G, et al. Albendazole treatment of children with ascariasis enhances the vibriocidal antibody response to the live attenuated oral cholera vaccine CVD 103-HgR. *J Infect Dis* 2000; **182**(4): 1199-206.
51. Bhuiyan TR, Choudhury FK, Khanam F, et al. Evaluation of immune responses to an oral typhoid vaccine, Ty21a, in children from 2 to 5 years of age in Bangladesh. *Vaccine* 2014; **32**(9): 1055-60.
52. Grassly NC, Praharaj I, Babji S, et al. The effect of azithromycin on the immunogenicity of oral poliovirus vaccine: a double-blind randomised placebo-controlled trial in seronegative Indian infants. *The Lancet Infectious Diseases* 2016; **16**(8): 905-14.
53. Hahn-Zoric M, Fulconis F, Minoli I, et al. Antibody responses to parenteral and oral vaccines are impaired by conventional and low protein formulas as compared to breast-feeding. *Acta Paediatrica Scandinavica* 1990; **79**(12): 1137-42.
54. John TJ, Devarajan LV, Luther L, Vijayarathnam P. Effect of breast-feeding on seroresponse of infants to oral poliovirus vaccination. *Pediatrics* 1976; **57**(1): 47-53.
55. Ali A, Kazi AM, Cortese MM, et al. Impact of withholding breastfeeding at the time of vaccination on the immunogenicity of oral rotavirus vaccine--a randomized trial.[Erratum appears in PLoS One. 2015;10(12):e0145568; PMID: 26673426]. *PLoS ONE [Electronic Resource]* 2015; **10**(6): e0127622.
56. Groome MJ, Koen A, Fix A, et al. Safety and immunogenicity of a parenteral P2-VP8-P[8] subunit rotavirus vaccine in toddlers and infants in South Africa: a randomised, double-blind, placebo-controlled trial. *Lancet Infect Dis* 2017; **17**(8): 843-53.
57. Rongsen-Chandola T, Strand TA, Goyal N, et al. Effect of withholding breastfeeding on the immune response to a live oral rotavirus vaccine in North Indian infants. *Vaccine* 2014; **32 Suppl 1**: A134-9.
58. Ahmed T, Svennerholm A-M, Al Tarique A, Sultana GNN, Qadri F. Enhanced immunogenicity of an oral inactivated cholera vaccine in infants in Bangladesh obtained by zinc supplementation and by temporary withholding breast-feeding. *Vaccine* 2009; **27**(9): 1433-9.
59. Chandir S, Ahamed KU, Baqui AH, et al. Effect of buffer on the immune response to trivalent oral poliovirus vaccine in Bangladesh: a community based randomized controlled trial. *Journal of Infectious Diseases* 2014; **210 Suppl 1**: S390-7.
60. Ceyhan M, Kanra G, Secmeer G, et al. Take of rhesus-human reassortant tetravalent rotavirus vaccine in breast-fed infants. *Acta Paediatrica* 1993; **82**(3): 223-7.
61. Clark HF, Burke CJ, Volkin DB, et al. Safety, immunogenicity and efficacy in healthy infants of G1 and G2 human reassortant rotavirus vaccine in a new stabilizer/buffer liquid formulation. *Pediatric Infectious Disease Journal* 2003; **22**(10): 914-20.
62. Ing DJ, Glass RI, Woods PA, et al. Immunogenicity of tetravalent rhesus rotavirus vaccine administered with buffer and oral polio vaccine. *American Journal of Diseases of Children* 1991; **145**(8): 892-7.
63. Kerdpanich A, Chokephaibulkit K, Watanaveeradej V, et al. Immunogenicity of a live-attenuated human rotavirus RIX4414 vaccine with or without buffering agent. *Human Vaccines* 2010; **6**(3): 26.
64. Sack DA, Shimko J, Sack RB, et al. Comparison of alternative buffers for use with a new live oral cholera vaccine, Peru-15, in outpatient volunteers. *Infection & Immunity* 1997; **65**(6): 2107-11.
65. Trach DD, Cam PD, Ke NT, et al. Investigations into the safety and immunogenicity of a killed oral cholera vaccine developed in Viet Nam. *Bulletin of the World Health Organization* 2002; **80**(1): 2-8.
66. Levine MM, Ferreccio C, Cryz S, Ortiz E. Comparison of enteric-coated capsules and liquid formulation of Ty21a typhoid vaccine in randomised controlled field trial. *Lancet* 1990; **336**(8720): 891-4.
67. Simanjuntak CH, Paleologo FP, Punjabi NH, et al. Oral immunisation against typhoid fever in Indonesia with Ty21a vaccine. *Lancet* 1991; **338**(8774): 1055-9.
68. John TJ. Immune response of neonates to oral poliomyelitis vaccine. *Br Med J (Clin Res Ed)* 1984; **289**(6449): 881.
69. Kompithra RZ, Paul A, Manoharan D, et al. Immunogenicity of a three dose and five dose oral human rotavirus vaccine (RIX4414) schedule in south Indian infants. *Vaccine* 2014; **32 Suppl 1**: A129-33.
70. Lanata CF, Black RE, del Aguila R, et al. Protection of Peruvian children against rotavirus diarrhea of specific serotypes by one, two, or three doses of the RIT 4237 attenuated bovine rotavirus vaccine. *Journal of Infectious Diseases* 1989; **159**(3): 452-9.
71. Madhi SA, Cunliffe NA, Steele D, et al. Effect of human rotavirus vaccine on severe diarrhea in African infants. *New England Journal of Medicine* 2010; **362**(4): 289-98.

72. Steele AD, Reynders J, Scholtz F, et al. Comparison of 2 different regimens for reactogenicity, safety, and immunogenicity of the live attenuated oral rotavirus vaccine RIX4414 coadministered with oral polio vaccine in South African infants. *Journal of Infectious Diseases* 2010; **202 Suppl**: S93-100.
73. Black RE, Levine MM, Ferreccio C, et al. Efficacy of one or two doses of Ty21a Salmonella typhi vaccine in enteric-coated capsules in a controlled field trial. Chilean Typhoid Committee. *Vaccine* 1990; **8**(1): 81-4.
74. Ferreccio C, Levine MM, Rodriguez H, Contreras R. Comparative efficacy of two, three, or four doses of TY21a live oral typhoid vaccine in enteric-coated capsules: a field trial in an endemic area. *Journal of Infectious Diseases* 1989; **159**(4): 766-9.
75. Dong DX, Hu XM, Liu WJ, et al. Immunization of neonates with trivalent oral poliomyelitis vaccine (Sabin). *Bull World Health Organ* 1986; **64**(6): 853-60.
76. Jain PK, Dutta AK, Nangia S, Khare S, Saili A. Seroconversion following killed polio vaccine in neonates. *Indian J Pediatr* 1997; **64**(4): 511-5.
77. Khare S, Kumari S, Nagpal IS, Sharma D, Verghese T. Oral polio vaccination in infants: beneficial effect of additional dose at birth. *Indian J Pediatr* 1993; **60**(2): 275-81.
78. Osei-Kwasi M, Afari EA, Mimura K, Obeng-Ansah I, Ampofo WK, Nkrumah FK. Randomized, controlled trial of trivalent oral poliovirus vaccine (Sabin) starting at birth in Ghana. *Bulletin of the World Health Organization* 1995; **73**(1): 41-6.
79. Weckx LY, Schmidt BJ, Herrmann AA, Miyasaki CH, Novo NF. Early immunization of neonates with trivalent oral poliovirus vaccine. *Bulletin of the World Health Organization* 1992; **70**(1): 85-91.
80. Bines JE, Danchin M, Jackson P, et al. Safety and immunogenicity of RV3-BB human neonatal rotavirus vaccine administered at birth or in infancy: a randomised, double-blind, placebo-controlled trial. *The Lancet Infectious Diseases* 2015; **15**(12): 1389-97.
81. Bhaskaram P, Balakrishna N. Effect of administration of 200,000 IU of vitamin A to women within 24 hrs after delivery on response to PPV administered to the newborn. *Indian Pediatrics* 1998; **35**(3): 217-22.
82. Domok I, Balayan MS, Fayinka OA, Skrtic N, Soneji AD, Harland PS. Factors affecting the efficacy of live poliovirus vaccine in warm climates. Efficacy of type 1 Sabin vaccine administered together with antihuman gamma-globulin horse serum to breast-fed and artificially fed infants in Uganda. *Bulletin of the World Health Organization* 1974; **51**(4): 333-47.
83. Ostrom KM, Cordle CT, Schaller JP, et al. Immune status of infants fed soy-based formulas with or without added nucleotides for 1 year: part 1: vaccine responses, and morbidity. *J Pediatr Gastroenterol Nutr* 2002; **34**(2): 137-44.
84. Wasserman SS, Kotloff KL, Losonsky GA, Levine MM. Immunologic response to oral cholera vaccination in a crossover study: a novel placebo effect. *American Journal of Epidemiology* 1993; **138**(11): 988-93.
85. Cohen-Abbo A, Culley BS, Reed GW, et al. Seroresponse to trivalent oral poliovirus vaccine as a function of dosage interval. *Pediatric Infectious Disease Journal* 1995; **14**(2): 100-6.
86. Estivariz CF, Anand A, Gary HE, Jr., et al. Immunogenicity of three doses of bivalent, trivalent, or type 1 monovalent oral poliovirus vaccines with a 2 week interval between doses in Bangladesh: an open-label, non-inferiority, randomised, controlled trial. *The Lancet Infectious Diseases* 2015; **15**(8): 898-904.
87. Mir F, Quadri F, Mach O, et al. Monovalent type-1 oral poliovirus vaccine given at short intervals in Pakistan: a randomised controlled, four-arm, open-label, non-inferiority trial. *The Lancet Infectious Diseases* 2015; **15**(8): 889-97.
88. Asturias EJ, Bandyopadhyay AS, Self S, et al. Humoral and intestinal immunity induced by new schedules of bivalent oral poliovirus vaccine and one or two doses of inactivated poliovirus vaccine in Latin American infants: an open-label randomised controlled trial. *Lancet* 2016; **388**(10040): 158-69.
89. el-Sayed N, el-Gamal Y, Abbassy AA, et al. Monovalent type 1 oral poliovirus vaccine in newborns. *N Engl J Med* 2008; **359**(16): 1655-65.
90. John TJ, Devarajan LV, Balasubramanyan A. Immunization in India with trivalent and monovalent oral poliovirus vaccines of enhanced potency. *Bull World Health Organ* 1976; **54**(1): 115-7.
91. Saleem AF, Mach O, Yousafzai MT, et al. Immunogenicity of Different Routine Poliovirus Vaccination Schedules: A Randomized, Controlled Trial in Karachi, Pakistan. *J Infect Dis* 2018; **217**(3): 443-50.
92. Schoub BD, Johnson S, McAnerney J, Gilbertson L, Klaassen KI, Reinach SG. Monovalent neonatal polio immunization--a strategy for the developing world. *J Infect Dis* 1988; **157**(4): 836-9.
93. Sutter RW, John TJ, Jain H, et al. Immunogenicity of bivalent types 1 and 3 oral poliovirus vaccine: a randomised, double-blind, controlled trial. *Lancet* 2010; **376**(9753): 1682-8.
94. Sutter RW, Suleiman AJ, Malankar P, et al. Trial of a supplemental dose of four poliovirus vaccines. *New England Journal of Medicine* 2000; **343**(11): 767-73.

95. Waggie Z, Geldenhuys H, Sutter RW, et al. Randomized trial of type 1 and type 3 oral monovalent poliovirus vaccines in newborns in Africa. *J Infect Dis* 2012; **205**(2): 228-36.
96. Broome CS, McArdle F, Kyle JA, et al. An increase in selenium intake improves immune function and poliovirus handling in adults with marginal selenium status. *Am J Clin Nutr* 2004; **80**(1): 154-62.
97. Taffs RE, Enterline JC, Rusmil K, et al. Oral iodine supplementation does not reduce neutralizing antibody responses to oral poliovirus vaccine. *Bull World Health Organ* 1999; **77**(6): 484-91.
98. Wolvers DAW, van Herpen-Broekmans WMR, Logman MHGM, van der Wielen RPJ, Albers R. Effect of a mixture of micronutrients, but not of bovine colostrum concentrate, on immune function parameters in healthy volunteers: a randomized placebo-controlled study. *Nutrition Journal* 2006; **5**: 28.
99. Isolauri E, Joensuu J, Suomalainen H, Luomala M, Vesikari T. Improved immunogenicity of oral D x RRV reassortant rotavirus vaccine by Lactobacillus casei GG. *Vaccine* 1995; **13**(3): 310-2.
100. Matsuda F, Chowdhury MI, Saha A, et al. Evaluation of a probiotics, Bifidobacterium breve BBG-01, for enhancement of immunogenicity of an oral inactivated cholera vaccine and safety: a randomized, double-blind, placebo-controlled trial in Bangladeshi children under 5 years of age.[Erratum appears in Vaccine. 2011 Aug 11;29(35):6068]. *Vaccine* 2011; **29**(10): 1855-8.
101. Paineau D, Carcano D, Leyer G, et al. Effects of seven potential probiotic strains on specific immune responses in healthy adults: a double-blind, randomized, controlled trial. *FEMS Immunology & Medical Microbiology* 2008; **53**(1): 107-13.
102. Fang H, Elina T, Heikki A, Seppo S. Modulation of humoral immune response through probiotic intake. *FEMS Immunol Med Microbiol* 2000; **29**(1): 47-52.
103. Zaman K, Sack DA, Yunus M, et al. Successful co-administration of a human rotavirus and oral poliovirus vaccines in Bangladeshi infants in a 2-dose schedule at 12 and 16 weeks of age. *Vaccine* 2009; **27**(9): 1333-9.
104. Ciarlet M, Sani-Grosso R, Yuan G, et al. Concomitant use of the oral pentavalent human-bovine reassortant rotavirus vaccine and oral poliovirus vaccine. *Pediatric Infectious Disease Journal* 2008; **27**(10): 874-80.
105. Giammanco G, De Grandi V, Lupo L, et al. Interference of oral poliovirus vaccine on RIT 4237 oral rotavirus vaccine. *Eur J Epidemiol* 1988; **4**(1): 121-3.
106. Hanlon P, Hanlon L, Marsh V, et al. Trial of an attenuated bovine rotavirus vaccine (RIT 4237) in Gambian infants. *Lancet* 1987; **1**(8546): 1342-5.
107. Migasena S, Simasathien S, Samakoses R, et al. Simultaneous administration of oral rhesus-human reassortant tetravalent (RRV-TV) rotavirus vaccine and oral poliovirus vaccine (OPV) in Thai infants. *Vaccine* 1995; **13**(2): 168-74.
108. Vodopija I, Baklaic Z, Vlatkovic R, Bogaerts H, Delem A, Andre FE. Combined vaccination with live oral polio vaccine and the bovine rotavirus RIT 4237 strain. *Vaccine* 1986; **4**(4): 233-6.
109. Agarwal A, Sharma D, Kumari S, Khare S. Antibody response to three doses of standard and double dose of trivalent oral polio vaccine. *Indian Pediatr* 1991; **28**(10): 1141-5.
110. Chopra K, Kundu S, Chowdhury DS. Antibody response of infants in tropics to five doses of oral polio vaccine. *Journal of Tropical Pediatrics* 1989; **35**(1): 19-23.
111. Factors affecting the immunogenicity of oral poliovirus vaccine: a prospective evaluation in Brazil and the Gambia. World Health Organization Collaborative Study Group on Oral Poliovirus Vaccine. *J Infect Dis* 1995; **171**(5): 1097-106.
112. Gotuzzo E, Butron B, Seas C, et al. Safety, immunogenicity, and excretion pattern of single-dose live oral cholera vaccine CVD 103-HgR in Peruvian adults of high and low socioeconomic levels. *Infect Immun* 1993; **61**(9): 3994-7.
113. Sow SO, Tapia MD, Chen WH, et al. A randomized, placebo-controlled, double-blind Phase 2 trial comparing the reactogenicity and immunogenicity of a single >/=2x10(8) colony forming units [cfu] standard-dose versus a >/=2x10(9) cfu high-dose of CVD 103-HgR live attenuated oral cholera vaccine, with Shanchol inactivated oral vaccine as an open label immunologic comparator. *Clin Vaccine Immunol* 2017.
114. Suharyono, Simanjuntak C, Witham N, et al. Safety and immunogenicity of single-dose live oral cholera vaccine CVD 103-HgR in 5-9-year-old Indonesian children. *Lancet* 1992; **340**(8821): 689-94.
115. Bahl R, Bhandari N, Kant S, Molbak K, Ostergaard E, Bhan MK. Effect of vitamin A administered at Expanded Program on Immunization contacts on antibody response to oral polio vaccine. *European Journal of Clinical Nutrition* 2002; **56**(4): 321-5.
116. Newton S, Cousens S, Owusu-Agyei S, et al. Vitamin a supplementation does not affect infants' immune responses to polio and tetanus vaccines. *Journal of Nutrition* 2005; **135**(11): 2669-73.
117. Rahman MM, Alvarez JO, Mahalanabis D, et al. Effect of vitamin a administration on response to oral pollo vaccination. *Nutrition Research* 1998; **18**(7): 1125-33.

118. Semba RD, Muhilal, Mohgaddam NE, et al. Integration of vitamin A supplementation with the expanded program on immunization does not affect seroconversion to oral poliovirus vaccine in infants. *Journal of Nutrition* 1999; **129**(12): 2203-5.
119. Mwanza-Lisulo M, Kelly P. Retinoic acid elicits a coordinated expression of gut homing markers on T lymphocytes with oral typhoid, but not viral, vaccines in Zambian men. *Gastroenterology* 2016; **150**(4): S386.
120. Habib MA, Soofi S, Sheraz A, et al. Zinc supplementation fails to increase the immunogenicity of oral poliovirus vaccine: a randomized controlled trial. *Vaccine* 2015; **33**(6): 819-25.
121. Ahmed T, Arifuzzaman M, Lebens M, Qadri F, Lundgren A. CD4+ T-cell responses to an oral inactivated cholera vaccine in young children in a cholera endemic country and the enhancing effect of zinc supplementation. *Vaccine* 2009; **28**(2): 422-9.
122. Karlsen TH, Sommerfelt H, Klomstad S, et al. Intestinal and systemic immune responses to an oral cholera toxoid B subunit whole-cell vaccine administered during zinc supplementation. *Infection & Immunity* 2003; **71**(7): 3909-13.
